# Use of Artificial Intelligence, Internet of Things, and Edge Intelligence in Long-Term Care for Older People: Comprehensive Analysis Through Bibliometric, Google Trends, and Content Analysis

**DOI:** 10.2196/56692

**Published:** 2025-03-04

**Authors:** Shuo-Chen Chien, Chia-Ming Yen, Yu-Hung Chang, Ying-Erh Chen, Chia-Chun Liu, Yu-Ping Hsiao, Ping-Yen Yang, Hong-Ming Lin, Tsung-En Yang, Xing-Hua Lu, I-Chien Wu, Chih-Cheng Hsu, Hung-Yi Chiou, Ren-Hua Chung

**Affiliations:** 1 Institute of Population Health Sciences National Health Research Institutes Miaoli County Taiwan; 2 National Center for Geriatrics and Welfare Research National Health Research Institutes Yunlin County Taiwan; 3 Graduate Institute of Biomedical Sciences China Medical University Taichung City Taiwan; 4 Department of Risk Management and Insurance Tamkang University New Taipei City Taiwan; 5 School of Public Health College of Public Health Taipei Medical University Taipei Taiwan

**Keywords:** bibliometric analysis, Google Trends, content analysis, long-term care, older adults, artificial intelligence, Internet of Things, edge intelligence

## Abstract

**Background:**

The global aging population poses critical challenges for long-term care (LTC), including workforce shortages, escalating health care costs, and increasing demand for high-quality care. Integrating artificial intelligence (AI), the Internet of Things (IoT), and edge intelligence (EI) offers transformative potential to enhance care quality, improve safety, and streamline operations. However, existing research lacks a comprehensive analysis that synthesizes academic trends, public interest, and deeper insights regarding these technologies.

**Objective:**

This study aims to provide a holistic overview of AI, IoT, and EI applications in LTC for older adults through a comprehensive bibliometric analysis, public interest insights from Google Trends, and content analysis of the top-cited research papers.

**Methods:**

Bibliometric analysis was conducted using data from Web of Science, PubMed, and Scopus to identify key themes and trends in the field, while Google Trends was used to assess public interest. A content analysis of the top 1% of most-cited papers provided deeper insights into practical applications.

**Results:**

A total of 6378 papers published between 2014 and 2023 were analyzed. The bibliometric analysis revealed that the United States, China, and Canada are leading contributors, with strong thematic overlaps in areas such as dementia care, machine learning, and wearable health monitoring technologies. High correlations were found between academic and public interest, in key topics such as “long-term care” (τ=0.89, *P*<.001) and “caregiver” (τ=0.72, *P*=.004). The content analysis demonstrated that social robots, particularly PARO, significantly improved mood and reduced agitation in patients with dementia. However, limitations, including small sample sizes, short study durations, and a narrow focus on dementia care, were noted.

**Conclusions:**

AI, IoT, and EI collectively form a powerful ecosystem in LTC settings, addressing different aspects of care for older adults. Our study suggests that increased international collaboration and the integration of emerging themes such as “rehabilitation,” “stroke,” and “mHealth” are necessary to meet the evolving care needs of this population. Additionally, incorporating high-interest keywords such as “machine learning,” “smart home,” and “caregiver” can enhance discoverability and relevance for both academic and public audiences. Future research should focus on expanding sample sizes, conducting long-term multicenter trials, and exploring broader health conditions beyond dementia, such as frailty and depression.

## Introduction

The global long-term care (LTC) sector faces significant challenges, primarily driven by an aging population and increasing demand for services [1]. This surge strains existing systems, exposing critical issues such as workforce shortages and the physically and emotionally demanding nature of LTC work [2,3]. Additionally, the rising costs associated with delivering high-quality care challenge the sustainability of current LTC models, emphasizing the urgent need for innovative and efficient solutions [4,5]. In this context, integrating technologies such as artificial intelligence (AI), the Internet of Things (IoT), and edge intelligence (EI) into LTC presents substantial advantages [6]. AI facilitates predictive health care by enabling timely interventions and personalized care strategies, while IoT devices such as sensors and wearables provide continuous health monitoring, enhancing resident safety and autonomy [7,8]. EI complements these technologies by enabling real-time decision-making through local data processing, reducing latency, and improving responsiveness in care settings [9,10]. Together, these not only improve care quality but also alleviate the workload of health care staff by automating routine tasks.

Numerous studies have investigated the adoption and understanding of these technologies through methods such as bibliometric analysis, Google Trends, and content analysis. Bibliometric analysis provides a quantitative evaluation of academic literature, highlighting key contributors, research trends, and emerging topics within a field [11]. Google Trends offers valuable insights into public interest and search behaviors, tracking the popularity of specific topics across regions over time [12]. Meanwhile, content analysis allows for a qualitative examination of thematic patterns and sentiment across textual data sources [13]. Representative examples are as follows: Maugeri et al [14] analyzed global public and research interest in telemedicine from 2017 to 2022 using Google Trends and bibliometric data, highlighting the impact of COVID-19 on interest and publication growth; Wang et al [15] mapped the global research landscape on AI applications in geriatrics using bibliometric analysis, identifying major contributors and key topics such as dementia and Alzheimer’s care. Additionally, Voleti and Bhat [16] conducted a bibliometric analysis focused on IoT and edge computing in health care, revealing the growing application of these technologies in smart health care, with significant challenges in data management and scalability. Similarly, Ziwei et al [17] performed a deep bibliometric study on IoT in smart health care, highlighting key trends such as the increasing integration of blockchain-based security and network connectivity in health care systems. However, while these studies provide valuable insights into specific aspects of these technologies, they often remain limited in scope by focusing solely on either bibliometric analysis, Google Trends, or content analysis. This fragmented approach may hinder a comprehensive understanding of the broader landscape. Therefore, there is a need for a more integrative overview that explores the combined impact of these advanced digital tools on LTC. By synthesizing bibliometric analysis, Google Trends, and content analysis, this study aims to bridge this gap and offer a more holistic perspective on how these smart systems are applied in LTC.

This paper seeks to address the identified gaps by offering a more comprehensive and integrated analysis of AI, IoT, and EI applications in LTC for older adults. By combining bibliometric analysis, Google Trends, and content analysis, the study provides both broader and deeper insights into the usage of these technologies. The bibliometric analysis approach maps the intellectual structure of the field, identifying key contributors and thematic areas, while Google Trends analysis reveals public interest in the same topics, expanding the scope of understanding. Content analysis of the top 1% of the most-cited, high-impact studies offers in-depth insights into the practical applications and challenges of these technologies in real-world LTC settings. This interdisciplinary approach not only enriches the academic discourse but also provides a clearer road map for future research and development in care technologies for older adults.

This study aims to comprehensively explore the integration of AI, the IoT, and EI in LTC for older adults, addressing diverse perspectives, including academic frameworks, public interest, and in-depth insights from high-impact research. Specifically, bibliometric analysis is used to map the intellectual structure of the field, identifying leading contributors, influential publications, and key research trends. Public interest is examined through Google Trends, offering insights into societal attention to relevant topics and their alignment with academic focus. Additionally, a detailed content analysis of the top 1% most-cited research papers is conducted to uncover practical applications and challenges associated with the deployment of these technologies in real-world LTC environments. By synthesizing these diverse methodological approaches, our study results could construct a comprehensive framework that enhances the understanding of current developments and informs future innovations in AI, IoT, and EI for improving the quality and efficiency of care for older adults.

## Methods

### Overview of the Research Process and Analytical Methods

**Figure 1:**
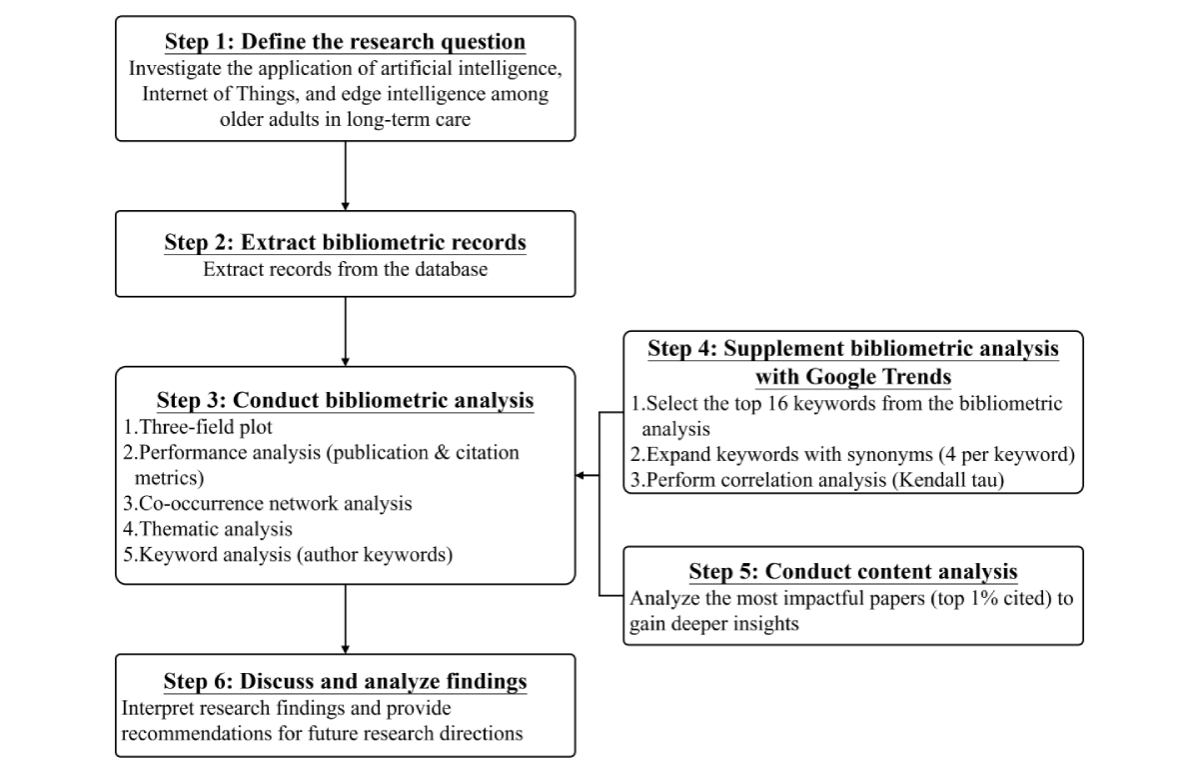
The overview of workflow in this study.

Figure 1 illustrates the complete research process undertaken in this study. The process begins with defining the research question, which focuses on investigating the application of AI, IoT, and EI among older adults in LTC. After establishing the research focus, relevant bibliometric records were extracted from the chosen databases. A comprehensive bibliometric analysis followed, encompassing the generation of a 3-field plot, performance analysis (based on publication and citation metrics), co-occurrence network analysis, thematic analysis, and keyword analysis to identify key research themes. To enhance the findings from the bibliometric analysis, a Google Trends analysis was conducted, where the top 16 keywords from the bibliometric analysis were expanded with synonyms. Kendall tau (τ) correlation analysis was performed to explore the relationship between keyword occurrences in bibliometric analysis and Google Trends data. Finally, a content analysis of the top 1% of the most-cited papers was conducted, offering deeper insights into practical applications. The results of these analyses inform the discussion and provide recommendations for future research directions.

For our analysis, we used the R package “Bibliometrix” (version 4.1.4; R Foundation) for bibliometric analysis, and Python 3.8 (Python Foundation) was used to conduct statistical analyses and create figures [18].

### Ethics Considerations

The data utilized in this research were sourced from publicly accessible repositories, qualifying as secondary data and negating the necessity for direct human participant engagement. Consequently, this study does not present any ethical concerns warranting approval from an institutional review board.

### The Extraction and Cleaning Process of Bibliometrics Data

We collected data through bibliographic searches across 3 major databases: Web of Science, PubMed, and Scopus. These searches, conducted in early September 2024, used a combination of keywords categorized into 3 groups: (1) terms about older adults; (2) terms linked to AI, IoT, or EI; and (3) terms associated with LTC, with the specific keywords detailed in Table S2 in Multimedia Appendix 1. Our inclusion criteria encompassed proceedings and early access papers, while we excluded papers published before 2013 or after 2023, non-English papers, and those classified under journal categories such as reviews, editorials, letters, books, corrections, and notes. The final search strategy used in each database is listed in Table S3 in Multimedia Appendix 1. Following recommendations from existing literature for ensuring robust bibliometric analysis, we set a minimum sample size of 200 papers for our qualitative review [19].

After the initial data extraction, we used Biblioshiny, a web-based application within the R Bibliometrix package designed for bibliometric analysis, to perform an automated data cleaning process aimed at improving data quality and reliability [18]. Duplicates arising from entries across multiple databases were identified and removed. To ensure consistency, we also standardized author affiliations, retaining only the most relevant affiliation for each author at the time of publication. This process minimized potential errors, such as incorrect or multiple affiliations, which could have affected the accuracy of the analysis. By implementing this cleaning procedure, we ensured that the final data set accurately represented the scholarly landscape.

### Bibliometric Analysis

This study used bibliometric analysis, a quantitative approach that leverages bibliographic data—such as authorship, citations, references, and keywords—to explore relationships and impacts within the field [11]. We focused on 5 distinct analysis methods to gain a comprehensive understanding of the intellectual structure of our research domain: 3-field plot, performance analysis (publication and citation metrics), co-occurrence network analysis, thematic analysis, and keyword analysis (author keywords). These methods are described in detail in the following sections.

### Overview Using the Three-Field Plot

To gain a comprehensive understanding of the intellectual landscape of our research field, we used a 3-field plot, a robust method for providing an insightful overview of the research cohort [20]. The 3-field plot, also known as a Sankey diagram, visually represents the flow and connections between different bibliometric dimensions. In this study, we integrated 3 critical dimensions: the geographic distribution of research (country), the dissemination mediums (journal), and the thematic concentration (keywords). This multidimensional approach is instrumental in mapping the intellectual territory of the research field, allowing us to visualize the relationships and transitions between where research is conducted, how it is disseminated, and the thematic focus of the studies.

### Performance Analysis Using Publication and Citation Numbers

To measure output performance, we used both the number of publications and citation counts [21]. The number of publications serves as a fundamental and credible metric for quantifying research output, systematically categorized by specific journals, institutions, or countries, providing a reliable measure of scholarly productivity and impact. Citation counts are a robust indicator of a paper’s influence within the scientific community, with higher citation counts suggesting that the paper’s findings have significantly supported subsequent research, reflecting increased scholarly interest and relevance in the field. Citation counts can be further categorized into 2 distinct metrics: total global citations (TGCs) and total local citations (TLCs). TGCs measure a paper’s citations across external publications, highlighting its global influence, while TLCs track citations within our curated data set, providing a focused view of its impact within a specific context. Additionally, the H-index was used to evaluate both the quantity and impact of scientific output, combining aspects of productivity and citation impact. This multifaceted approach, encompassing publication counts, global and local citations, and the H-index, offers a comprehensive view of scientific contributions and their influence within our research domain.

### Co-Occurrence Network Analysis

We used a co-occurrence network analysis, a methodological tool designed to visually map the interrelationships among various elements in scholarly literature [22]. This technique is particularly effective for illustrating connections formed through the simultaneous occurrence of elements such as keywords, authors, references, or terms in academic papers. In this study, we focused on analyzing the relationships among countries, institutions, and author’s keywords. In the co-occurrence network, each element is represented as a node, with connections (edges) drawn between nodes that frequently appear together in the same articles or contexts. The strength of these connections is determined by the frequency of co-occurrence; more frequent appearances result in thicker, more pronounced links. By examining these networks, researchers can identify clusters of interrelated works or concepts, revealing patterns and intellectual currents that shape academic research. Specifically, the clusters identified through the co-occurrence network of the author’s keywords provided the foundation for subsequent thematic mapping, where these clusters were further analyzed to explore thematic concentration and development within the research domain.

### Thematic Analysis

Thematic analysis is a method used to identify and interpret patterns (themes) within data, particularly the research themes emerging from bibliometric data. It categorizes and maps research themes based on 2 dimensions: Relevance degree (Centrality), which measures a theme’s importance within the research field, and Development degree (Density), which reflects how well-developed a theme is. The results are visually represented in Figure S5 in Multimedia Appendix 1, with themes plotted across 4 quadrants:

- Quadrant I (Motor Themes): Highly relevant and well-developed themes, central to the field and influential in driving research.
- Quadrant II (Niche Themes): Well-developed but less central themes, often specialized within narrower areas of study.
- Quadrant III (Emerging or Declining Themes): Less relevant and underdeveloped themes, either emerging trends or topics in decline.
- Quadrant IV (Basic Themes): Highly relevant but less developed themes that provide a foundation for broader research.

We divided our analysis into 3 phases, 2014-2016, 2017-2020, and 2021-2023, to observe how research themes have evolved. This approach allowed us to track shifts in focus, identify emerging trends, and highlight themes that have remained consistently relevant throughout the periods.

### Keyword Analysis Using Author Keywords

In this study, we performed a keyword analysis using author keywords, which are terms selected by authors to encapsulate the core themes of their research papers. These keywords are essential for identifying major research trends and understanding focus areas within a field, as they reflect the concepts scholars consider most important in their studies. We selected the 16 most frequently occurring author keywords from our data set and analyzed their yearly trends to track their prominence in academic research over time. This analysis provided insights into which topics gained or lost attention, highlighting shifts in research priorities. To further explore the relevance of these keywords beyond academia, we used Google Trends to examine public interest in the same 16 keywords, allowing us to compare academic trends with public search behavior and gain a broader understanding of the societal impact of these research themes.

### Enhancing Bibliometric Analysis With Public Interest Insights From Google Trends

We incorporated Google Trends as an analytical tool to capture public interest in the research topics identified through our keyword analysis [12]. Google Trends provides search volume scores ranging from 0 to 100 for each keyword, with 100 representing peak popularity, 50 indicating half of the peak popularity, and 0 reflecting insufficient data. The keywords selected for the Google Trends analysis were based on the most frequently occurring keywords from the bibliometric analysis, with the top 16 most frequent ones being chosen. To capture broader variations in public interest, we consulted with experts and selected 4 synonyms for each of these keywords, thereby expanding the search scope. The searches were conducted globally, covering the period from January 1, 2014, to December 31, 2023, with data provided on a monthly basis. We then calculated the annual search interest by averaging the monthly data for each year. Additionally, a weighted average of the search volumes for each keyword and its synonyms was computed to represent the overall Google Trends search interest for each term.

To align the units of comparison between academic keyword trends (from bibliometric analysis) and public interest (from Google Trends), we applied min-max scaling. This scaling was done globally across the 16 keywords and the period from 2014 to 2023, with the maximum and minimum values for both data sets used to normalize the data. After scaling, we multiplied each result by 100, ensuring that every value falls within the range of 0-100, enabling direct comparison between academic research frequency and public search volume. Finally, we applied the Kendall tau correlation coefficient, a nonparametric statistic, to measure the ordinal association between academic focus and public interest by comparing concordant and discordant pairs [23]. The correlation coefficient ranges from –1 (perfect inverse correlation) to +1 (perfect direct correlation), with *P*<.05 considered statistically significant. This method allowed us to quantitatively assess how well academic keyword trends aligned with public search interest over time.

### Content Analysis and Quality Assessment

To gain a deeper understanding of the research focus in this study, we conducted a content analysis on the most impactful studies, specifically those with higher TLCs. Content analysis is a qualitative research method used to systematically classify and interpret text data by identifying themes and patterns [13]. This approach allowed us to explore key terms, concepts, and themes related to AI and IoT applications in LTC, helping to uncover trends, patterns, and gaps in the existing research.

Before undertaking the content analysis, a quality assessment was performed to evaluate the methodological rigor of the papers included in our review. We used the Mixed Methods Appraisal Tool to assess the quality of the selected studies (Table S4 in Multimedia Appendix 1). This tool is designed to evaluate a wide range of study types, including qualitative studies, randomized controlled trials, nonrandomized quantitative studies, quantitative descriptive studies, and mixed methods research. Each study was assessed using a nominal scale (yes/no/cannot tell), ensuring that only high-quality papers were included in the content analysis. Two independent reviewers (SCC and CCL) carried out the quality assessment, with discrepancies ultimately resolved by the corresponding author (RHC).

For the content analysis, we selected the top 1% of papers from the overall cohort, ranked primarily by TLC and secondarily by TGC. This selection process ensured that the studies analyzed not only had substantial local impact within the field but also possessed significant global relevance. From these papers, we extracted key information, including the country or region of the corresponding author, whether the study was conducted at a single center or multiple centers, the total number of participants, the percentage of female participants, the illness type of participants, whether assistive robots were used, the study’s primary objective, main findings, and stated limitations. Based on this analysis, we gained deeper insights and provided recommendations for future research directions to address the identified gaps and challenges.

## Results

### Data Collection and Screening Process for Bibliometric Analysis

**Figure 2:**
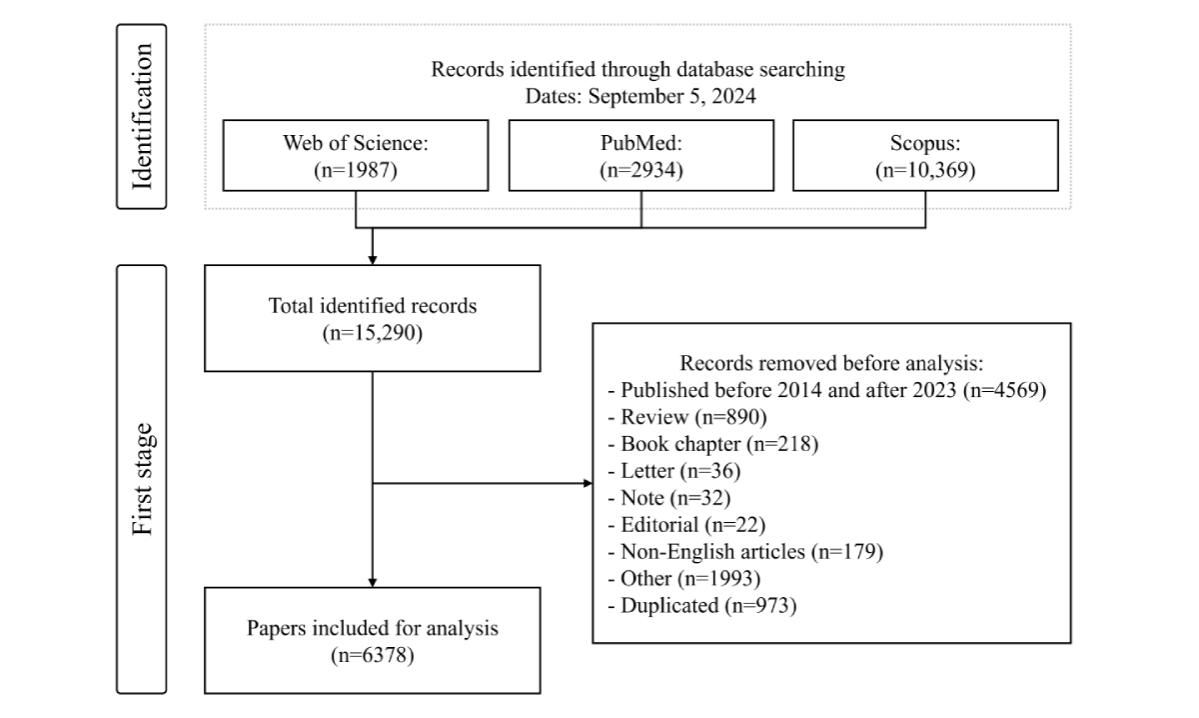
The procedure involved in choosing appropriate papers.

The keyword search across Web of Science, PubMed, and Scopus yielded 1987, 2934, and 10,369 records, respectively (Figure 2). Our screening process excluded papers published before 2014 or after 2023, resulting in 4569 eliminations. Additionally, we removed 890 review papers, 218 book chapters, 36 letters, 32 notes, 22 editorial materials, and 1993 other types of documents. Furthermore, 179 non-English papers were excluded. An additional 973 papers were discarded due to duplication. Ultimately, 6378 papers were selected for bibliometric analysis in the subsequent step. We also present the distribution of yearly publications and the average TGC per paper in Figure S6 in Multimedia Appendix 1.

### An Overview of This Study Field

**Figure 3:**
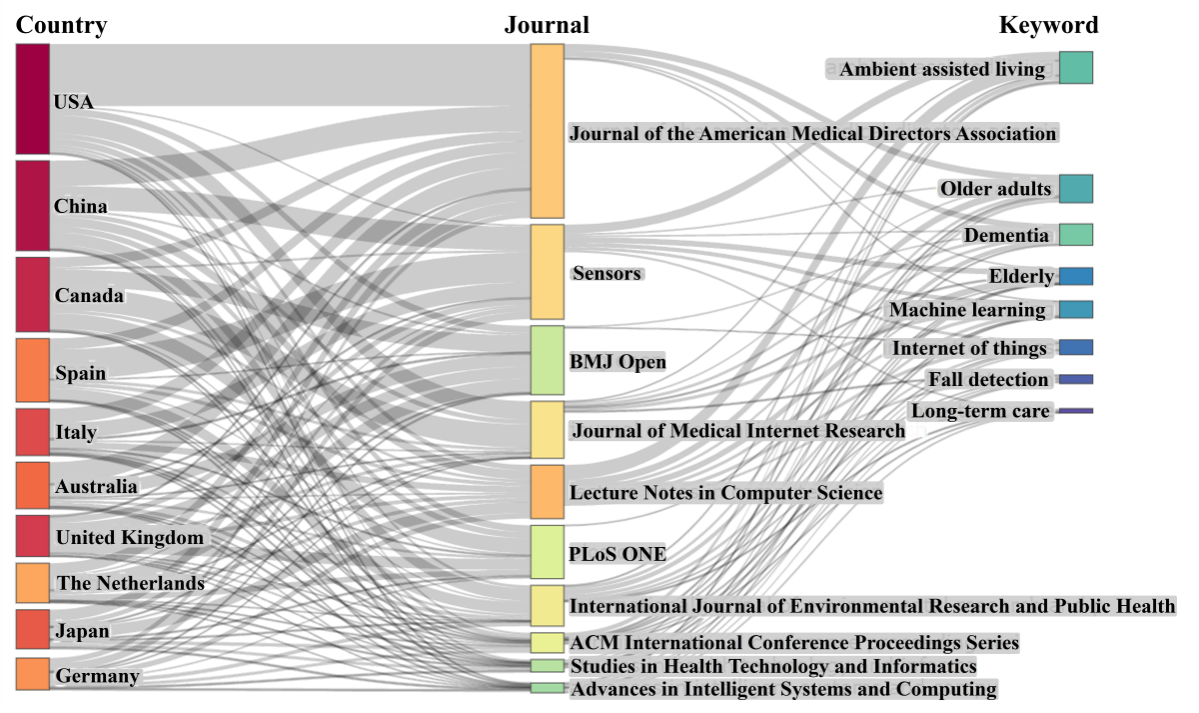
Three-field plot (country, journal, and keyword).

We used a Sankey diagram to analyze and illustrate the distribution and interplay among countries, journals, and keywords in the articles. This method effectively highlights the usage of AI, IoT, and EI among older adults in LTC across various countries (Figure 3).

In terms of country contributions, the United States leads with 291 articles, followed by China (n=238), Canada (n=197), Spain (n=168), and Italy (n=124). Other notable contributors include Australia (n=123), the United Kingdom (n=109), the Netherlands (n=105), Japan (n=103), and Germany (n=84). As for journals, the Journal of the American Medical Directors Association stands out with 460 articles, followed by Sensors (n=250), BMJ Open (n=182), and the Journal of Medical Internet Research (n=151). Other key journals include Lecture Notes in Computer Science (n=142), PLoS ONE (n=141), and the International Journal of Environmental Research and Public Health (n=108). These journals play a critical role in disseminating research on AI, IoT, and EI applications in LTC for older adults. Regarding keywords, prominent terms include “ambient assisted living” (n=84), “older adults” (n=74), “dementia” (n=57), and “elderly” (n=45). Other frequently used keywords are “machine learning” (n=45), “internet of things” (n=39), “fall detection” (n=23), and “long-term care” (n=12), reflecting the research focus on technological solutions aimed at improving the care and well-being of older adults.

The relationships between countries and journals reveal that major contributors such as the United States, China, and Canada publish across a wide range of journals, demonstrating a broad scope of research dissemination. Regarding journal-keyword relationships, distinct focus areas become apparent. For instance, the Journal of the American Medical Directors Association predominantly emphasizes studies on older adults and dementia, while Sensors focuses on topics such as ambient assisted living, machine learning, and care for older adults. By contrast, BMJ Open addresses general studies related to dementia and the IoT without a distinct keyword focus on other specific areas.

### Most Productive Journals and Institutions

Table 1 presents a detailed overview of the most productive journals publishing research related to AI, IoT, and EI in LTC for older adults, focusing on key metrics such as publication counts, TGC, and H-Index. Leading the list, Lecture Notes in Computer Science plays an important role in this domain, with 223 publications (N=6378, 3.50%) and a TGC of 1217. The Journal of the American Medical Directors Association, which specializes in geriatrics, aging, and LTC, follows closely with 113 publications (N=6378, 1.77%) and an impressive TGC of 5812. Meanwhile, Sensors, a journal primarily dedicated to technology and sensor applications in health care and IoT, has contributed 108 publications and a TGC of 2560. The Journal of Medical Internet Research, focused on digital health and eHealth technologies, adds 89 publications with a TGC of 1822. Notably, the International Journal of Social Robotics, which focuses on robotics and its applications in social settings, has a smaller output of 36 publications, but its TGC of 1471 highlights its substantial impact per article.

**Table 1:** Most influential journals (sorted by the number of publications; N=6378).

Ranking	Journals	Item, n (%)	Total global citations	Total global citations per item	H-Index
1	Lecture Notes in Computer Science	223 (3.50)	1217	5.46	15
2	Journal of the American Medical Directors Association	113 (1.77)	5812	51.43	32
3	Sensors	108 (1.69)	2560	23.70	25
4	Journal of Medical Internet Research	89 (1.40)	1822	20.47	24
5	International Journal of Environmental Research and Public Health	85 (1.33)	773	9.09	15
6	ACM International Conference Proceedings Series	80 (1.25)	339	4.24	11
7	PLoS ONE	77 (1.21)	1447	18.79	20
8	BMJ Open	68 (1.07)	912	13.41	15
9	Studies in Health Technology and Informatics	62 (0.97)	336	5.42	10
10	Advances in Intelligent Systems and Computing	51 (0.80)	216	4.24	6
11	BMC Geriatrics	51 (0.80)	911	17.86	15
12	Gerontechnology	51 (0.80)	133	2.61	5
13	CEUR Workshop Proceedings	45 (0.71)	101	2.24	6
14	IEEE Access	41 (0.64)	624	15.22	12
15	JMIR mHealth and uHealth	40 (0.63)	883	22.08	18
16	Lecture Notes in Electrical Engineering	39 (0.61)	60	1.54	5
17	JMIR Aging	38 (0.60)	413	10.87	12
18	International Journal of Social Robotics	36 (0.56)	1471	40.86	20
19	Journal of the American Geriatrics Society	34 (0.53)	755	22.21	18
20	Communications in Computer and Information Science	32 (0.50)	54	1.69	4

The H-Index further underscores the influence of these journals in the field. The Journal of the American Medical Directors Association stands out with a robust H-Index of 32, reflecting its high citation impact. Sensors and Lecture Notes in Computer Science follow with H-Indices of 25 and 15, respectively, illustrating their diverse influence within the field. The Journal of Medical Internet Research also demonstrates substantial scholarly impact, with an H-Index of 24. Finally, despite its smaller publication count, the International Journal of Social Robotics achieves an impressive H-Index of 20, underscoring the considerable influence of its limited number of publications.

We also identified the 20 most productive institutions worldwide based on their publication output, as detailed in Table S5 in Multimedia Appendix 1. Leading the list are the University of Toronto with 246 publications, the University of California with 169 publications, and the University of Michigan with 136 publications. Following closely are the University of Washington (n=120), Griffith University (n=106), and the University of Pittsburgh (n=102). Our analysis of the top 20 institutions reveals a diverse range of geographic locations, including institutions from Canada, the United States, Australia, the United Kingdom, Sweden, and Denmark. This global distribution underscores the widespread interest and active involvement in this field. The broad geographic representation not only highlights the substantial contributions of these institutions but also offers insights into potential collaboration opportunities across different regions.

### Co-Occurrence Network and Thematic Analysis

**Figure 4:**
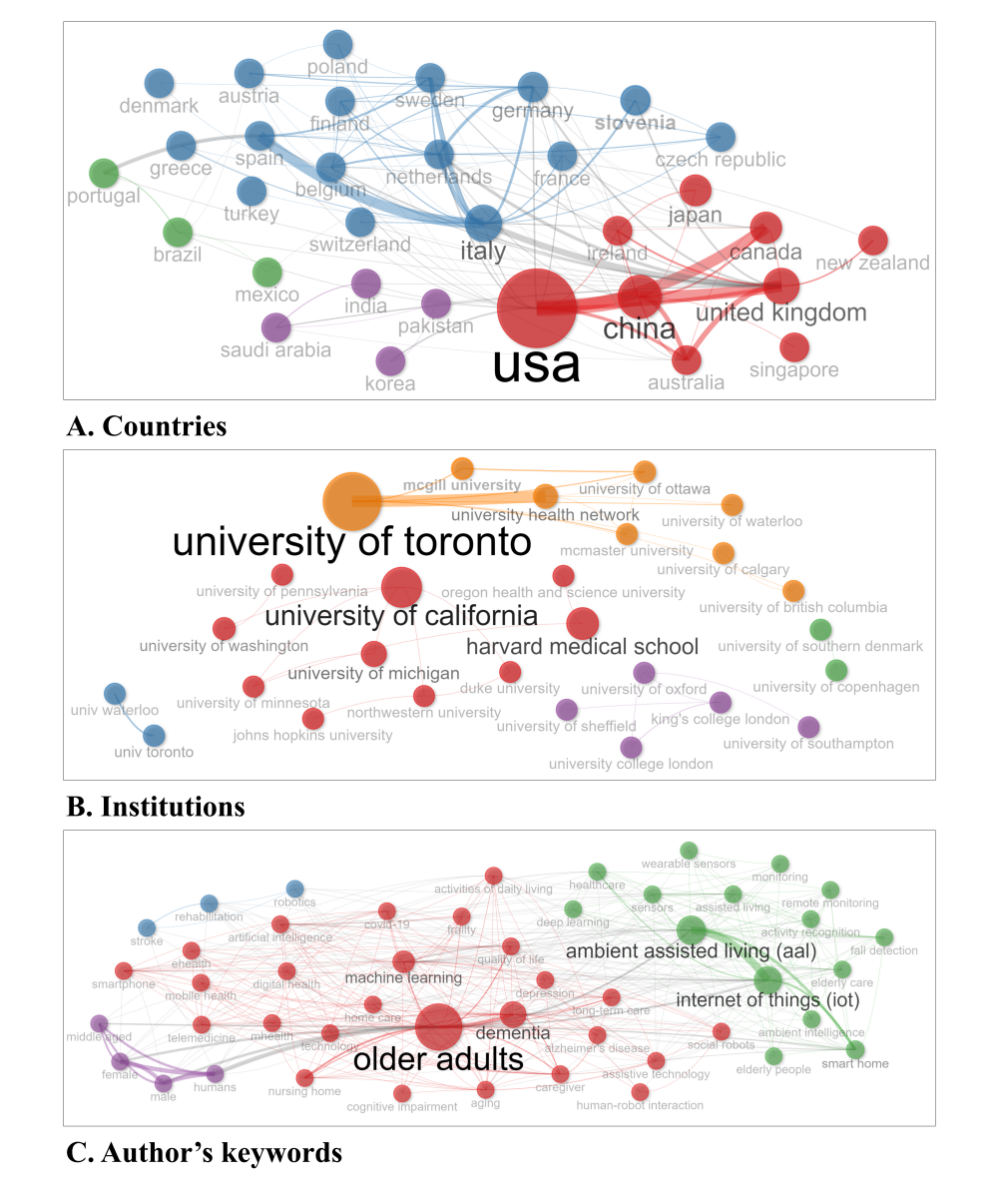
Co-occurrence network with three different fields.

In the bibliometric analysis, we constructed a co-occurrence network to explore the relationships and thematic intersections across 3 distinct fields: countries, institutions, and author’s keywords, covering the period from 2014 to 2023. As shown in Figure 4, the network highlights the complex interactions within the research landscape, delineating patterns of collaboration and thematic focus. The figure visually represents the prominence and centrality of specific countries, institutions, and keywords, revealing key players and dominant themes in the field. This network provides valuable insights into how different entities contribute to and shape research on AI, IoT, and EI in LTC for older adults.

Regarding the countries, the bibliometric network analysis reveals a robust international collaboration framework within the research landscape, as depicted in Figure 4A. The United States is prominently positioned at the core of this network, underscoring its pivotal role in spearheading research initiatives on a global scale. Notable are the substantial bilateral links connecting the United States with other research-heavy nations such as China, the United Kingdom, and Canada, which suggest a high degree of cooperative research activity. European countries, represented by the cohesive cluster of Germany, Italy, and France, demonstrate strong intracontinental connections, indicative of their collective research endeavors. Meanwhile, countries such as Portugal and Brazil, though less central in the visualization, reveal their targeted and specialized contributions to the research community.

The visualization of institutional collaborations (Figure 4B) positions the University of Toronto as a central node within the network, highlighting its significant research output and influence across disciplines. Other institutions, such as the University of California and Harvard Medical School, also command prominent places within the network, signifying their crucial roles in fostering extensive academic partnerships and knowledge dissemination. The depiction of a wide array of institutions from various corners of the globe points to a richly diverse and interdisciplinary academic milieu, signifying the breadth and depth of research collaborations and intellectual exchange.

The network of the author’s keywords (Figure 4C) is dominated by the term “older adults,” which acts as a thematic epicenter linking diverse research topics. Surrounding this pivotal term, we find noticeable nodes such as “dementia,” “health care,” and “assistive technology,” reflecting concentrated areas of research focus and societal impact. The prevalence of cutting-edge technological terms such as “artificial intelligence,” “internet of things,” and “machine learning” adjacent to care topics for older adults underscores the integration of technology in addressing the complexities of aging. Additionally, emergent terms such as “COVID-19,” “digital health,” and “telemedicine” are a testament to the field’s responsiveness to evolving global health issues and the increasing relevance of digital innovation in health care solutions.

To further understand the evolving research landscape, we conducted a thematic analysis of author keywords across 3 distinct periods: 2014-2016, 2017-2020, and 2021-2023. This analysis allowed us to track the shifts in research focus over the years and identify emerging or declining trends. The results, detailed and thoroughly documented in Multimedia Appendix 1 (see the section titled “Using Thematic Analysis for a Meticulous Examination Across Three Periods”) show noticeable changes in central research themes such as “older adults,” “dementia,” and “nursing home,” alongside the rise of newer themes such as “mHealth,” “digital health,” and “artificial intelligence” in recent years (see Figure S1 in Multimedia Appendix 1). These findings provide insights into how the integration of advanced technologies has increasingly shaped the research landscape in care for older adults, reflecting broader societal and health care trends.

### Keyword Analysis: Enhancing Bibliometric Analysis With Google Trends for the Top 16 Keywords

We also adopted the keywords analysis to explore the evolving trends and key focus areas in AI, IoT, and EI applications for older adults in LTC. In our study, we first identified the top 16 most frequently occurring keywords from the bibliometric analysis between 2014 and 2023. To further enhance the insights gained, we assessed public interest in these keywords using Google Trends. The detailed results of the keyword analysis, both from bibliometric analysis and Google Trends, are presented in Multimedia Appendix 1 under the section titled “Top 16 Frequent Keywords From Bibliometric Analysis and Their Google Trends Search Interest” (also see Table S1 and Figures S2-S4 in Multimedia Appendix 1). Among the most notable findings, “older adults” ranked as the most frequent keyword in the bibliometric analysis with 733 occurrences, and it consistently showed high search interest in Google Trends, peaking at 65 in 2020. Similarly, “machine learning” appeared 287 times in the bibliometric analysis and saw a notable rise in public interest, with its search score increasing from 15 in 2015 to 95 in 2023. The term “internet of things,” which occurred 353 times in the bibliometric analysis, also showed a modest growth in public interest, with its score rising from 39 in 2014 to 46 in 2023. By contrast, “ambient assisted living” peaked at 59 occurrences in the bibliometric analysis in 2017, but its search interest declined from 40 in 2017 to 18 in 2023. Additionally, “fall detection” exhibited lower public interest in the first 5 years, beginning to grow slowly in 2019 but remaining relatively steady over the last 5 years, despite being mentioned 152 times in the bibliometric analysis.

**Figure 5:**
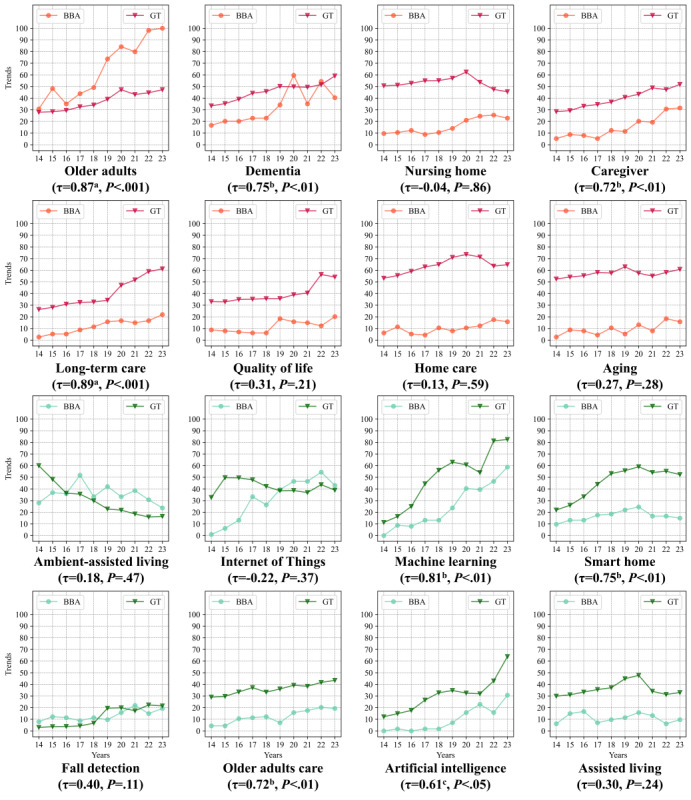
Correlation between bibliometric analysis and Google Trends volume.

Next, we analyzed the relationship between the keyword results from bibliometric analysis and Google Trends by calculating the correlation using the Kendall rank correlation coefficient. To address differences in scale between the 2 data sets, we applied min-max scaling to normalize the values into a range of 0-100 before conducting the analysis. The results are shown in Figure 5, and the statistical outcomes of this comparison are detailed in Table S6 in Multimedia Appendix 1. In the figure, the red trend lines represent keywords related to the older adults category. Among these, “long-term care” showed the strongest positive correlation (τ=0.89, *P*<.001), followed by “older adults” (τ=0.87, *P*<.001), both of which had significant positive correlations (*P*<.05), indicating a strong alignment between academic and public interest. The terms “dementia” (τ=0.75, *P*=.003) and “caregiver” (τ=0.72, *P*=.004) also exhibited significant positive correlations, reflecting growing public attention in these areas.

For the green trend lines, which represent keywords related to the ambient assisted living category, the strongest significant correlation was observed for “machine learning” (τ=0.81, *P*=.001), indicating increasing public and research interest. This was followed by “smart home” with a significant positive correlation (τ=0.75, *P*=.003). Similarly, “older adults care” showed a significant positive correlation (τ=0.72, *P*=.004), while “artificial intelligence” exhibited a moderate correlation (τ=0.61, *P*=.02). However, terms such as “fall detection” (τ=0.40, *P*=.11) and “ambient assisted living” (τ=0.18, *P*=.47) did not demonstrate statistically significant correlations, indicating weaker public interest compared with academic research in these areas.

### Content Analysis

To gain a deep understanding of the papers, we conducted a content analysis of the top 1% of most impactful papers, ranked primarily by TLC and secondarily by TGC, totaling 64 papers [24-87]. The quality assessment outcomes using the Mixed Methods Appraisal Tool are listed in Table S7 in Multimedia Appendix 1, along with the reasons. For the content analysis, we extracted key information such as the country or region of the corresponding author, whether the study was conducted at a single center or multiple centers, the total number of participants, the percentage of female participants, the illness type of participants, whether assistive robots were used, the study’s primary objective, main findings, and stated limitations in Table S8 in Multimedia Appendix 1.

The content analysis of the top 1% of impactful papers reveals key trends in the use of assistive technologies, particularly social robots, in care for older adults, especially for individuals with dementia. Many studies, such as those involving the robot PARO, consistently highlight its positive impact on reducing agitation and depression, and improving mood among patients with dementia. Long-term benefits were observed in several cases, with the psychosocial environment playing a critical role in the success of these interventions. Despite the overall positive outcomes, limitations such as small sample sizes, short study durations, and difficulties in maintaining participant engagement across various care settings were frequently noted.

A recurring theme is the exploration of the human-robot interaction and how it shapes care outcomes. For instance, multiple studies compared the effectiveness of robotic pets such as PARO against plush toys or live animals. These studies found that robots generally outperformed nonrobotic alternatives in enhancing engagement and managing symptoms. However, some studies, such as the one by Thodberg et al [53], noted a diminishing novelty effect over time, with live animals often prompting more sustained interaction. This highlights the need for future work to focus on maintaining long-term engagement with robotic interventions.

There is a significant focus on addressing the emotional and cognitive needs of older adults, especially those with mild cognitive impairment or more advanced dementia. Robots such as RobuLAB and CuDDler demonstrated potential in supporting cognitive and social functions, though customization and usability were critical factors influencing their acceptance. For example, Pino et al [39] highlighted how older adults and informal caregivers appreciated socially assistive robots for their adaptability, emphasizing the importance of designing robots that can be personalized to meet individual needs. Nevertheless, limitations such as small sample sizes and lack of direct interaction in some studies restricted generalizability.

Table 2 outlines key research gaps and future directions. A major limitation across studies is the absence of long-term follow-up, which constrains understanding of the sustained impacts of these technologies. Furthermore, the small sample sizes limit the generalizability of results. The narrow focus on dementia care, with limited exploration of other conditions such as frailty or depression, represents another critical gap. Lastly, there is insufficient research on the real-world implementation and scalability of these technologies. Future studies should prioritize long-term investigations, expand sample sizes through multicenter collaborations, broaden the scope to include other health conditions, and focus on real-world applicability, particularly scalability and cost-effectiveness.

**Table 2:** The summary of current research gaps and suggestions.

Current research gap	Suggestion
Studies focus on short-term outcomes.	Conduct longitudinal studies to assess long-term impact.
Small sample sizes limit generalizability.	Increase sample sizes through multicenter studies.
Research mostly targets dementia care.	Expand research to include conditions such as frailty and depression.
Limited real-world implementation and scalability data.	Prioritize studies in real-world settings and assess scalability and cost-effectiveness.

## Discussion

### Principal Findings

In this study, we utilized bibliometric analysis, Google Trends, and content analysis to gain a comprehensive understanding of the research interest in AI, IoT, and EI applications for older adults in LTC over the last decade. Our bibliometric analysis results included 6378 papers that revealed geographic diversity, with leading contributions from countries like the United States, China, and Canada and institutions such as the University of Toronto and the University of California. The increasing focus on keywords such as “older adults,” “machine learning,” and “internet of things” highlights their growing importance in enhancing care delivery. Additionally, the convergence of societal and research interests is reflected in the strong correlations between “long-term care,” “dementia,” and “caregiver” in both bibliometric analysis and Google Trends. Content analysis of the most impactful papers demonstrated that assistive technologies, particularly social robots such as PARO, consistently benefit patients with dementia by improving mood and reducing agitation. Overall, AI, IoT, and EI show significant potential in transforming personalized care, safety, and decision-making in care settings for older adults.

### Implications

#### Artificial Intelligence: The Usage in LTC

AI’s potential in the care of older adults is profoundly impactful, particularly in enhancing personalized care and engagement for older adults. Social robots, such as PARO, exemplify how AI can address the emotional and psychological needs of older adults, especially those with dementia. In Norway, a study involving 60 participants found that the use of PARO significantly reduced agitation and depression among patients with dementia, with positive effects lasting up to 3 months after intervention [34]. Similarly, in Australia, a larger study with 415 participants demonstrated that PARO fostered higher engagement and improved mood when compared with a plush toy used in control settings [61]. These results suggest that AI-powered robots can simulate companionship and therapeutic interactions, providing psychological comfort and enhancing the quality of life in LTC facilities. Furthermore, AI’s adaptability enables tailored interventions, where robots such as PARO adjust their responses based on real-time emotional cues, offering a more personalized and human-like experience for older residents. As such, AI is not merely a tool for automation but a transformative technology in care for older adults, contributing to improved mental health, reduced reliance on pharmacological interventions, and enhanced social interaction.

#### Internet of Things: The Impact on Health Monitoring and Safety in LTC

IoT technologies are revolutionizing safety and continuous health monitoring in older adult care settings by providing real-time data and immediate interventions. Wearable devices and sensors are increasingly used to monitor vital signs such as heart rate, movement, and sleep patterns, significantly contributing to the well-being of older adults. For example, a Hong Kong study developed an Internet of Medical Things system that monitored real-time biometric data, enabling health care providers to proactively modify care plans based on continuous monitoring [83]. This system demonstrated improved operational efficiency in care facilities, reducing the likelihood of health crises such as falls or cardiovascular events. Additionally, IoT solutions such as fall detection systems have been widely adopted in care facilities to enhance resident safety. Studies have shown that IoT-enabled wearable sensors effectively track motor activity and detect falls in real time, thus preventing serious injuries by alerting staff immediately [27,28,57]. These technologies not only improve patient outcomes but also alleviate the burden on caregivers by providing constant, automated oversight. By promoting greater independence among older residents, IoT devices allow them to age in place, contributing to both their safety and quality of life.

#### Edge Intelligence: Enhancing Real-Time Decision-Making in LTC

EI plays a pivotal role in enhancing care for older adults by enabling the processing of data directly at the source, thus reducing latency and ensuring real-time decision-making. EI allows care facilities to make immediate adjustments to care plans based on real-time data, optimizing both AI and IoT applications in care for older adults. For example, in an Italian study, a radar smart sensor utilizing edge computing successfully detected falls and monitored vital signs in older adults, providing immediate feedback to caregivers [57]. This approach minimizes the delay associated with cloud-based systems, ensuring that critical health decisions are made without the need for external data processing. Another study integrated EI into an IoT-based monitoring system, which facilitated the detection of behavioral changes in older adult residents, enabling care providers to make real-time interventions to prevent health deterioration [77]. By processing data locally, EI enhances the reliability and responsiveness of care systems, reducing the risk of system failures or connectivity issues. Moreover, EI ensures the security of sensitive health data by limiting the amount of personal information transmitted over networks, thus addressing privacy concerns that are prevalent in older adult care technologies.

### Summary and Suggestions for Future Research

AI, IoT, and EI collectively form a powerful ecosystem in LTC settings, addressing different aspects of care for older adults. Based on our results, we propose the following suggestions for future research directions. **First**, the co-occurrence network shows that this research topic is globally recognized, with various countries leading relevant studies. We recommend continuing to enhance international collaboration. The thematic map reveals emerging trends in “rehabilitation,” “stroke,” “robotics,” “caregiver,” “mHealth,” and “digital health.” Future research should focus on integrating these themes into broader studies to address the evolving needs in care for older adults. In addition, keyword analysis shows increasing interest in areas such as “older adults,” “machine learning,” and “internet of things.” The correlation of keywords between bibliometric analysis and Google Trends, including “long-term care,” “machine learning,” “dementia,” and “smart home,” reflects alignment between academic and public interest. Thus, we suggest that future research papers could enhance their discoverability by incorporating these keywords, ensuring they resonate with both scholarly communities and the general public. Finally, content analysis shows the potential of assistive technologies, particularly social robots such as PARO, in improving mood and reducing agitation among patients with dementia. Future studies should address limitations such as small sample sizes and short study durations by conducting larger, multicenter trials that examine long-term impacts. Expanding research to cover a wider range of health conditions, such as frailty or depression, would provide a more holistic understanding of how AI, IoT, and EI technologies can transform older adult care.

### Comparison With Previous Studies

Previous studies have consistently demonstrated the role of AI and IoT in care for older adults, especially in addressing cognitive impairments such as dementia. Wang et al’s [15] bibliometric analysis identified the United States as a leading contributor to AI applications in care for older adults, particularly in areas such as Alzheimer disease and dementia. Our study corroborates Wang et al’s findings by also highlighting a strong focus on AI and IoT for these conditions; however, we provide a more comprehensive analysis, evaluating over 6000 publications compared with Wang et al’s 230. Similarly, the study by Loveys et al [88] explored the acceptance of AI-augmented social robots and sensors, noting their varied effectiveness across care settings. Our findings align with Loveys et al’s observations, especially in high-income facilities where technologies such as PARO show greater efficacy. Additionally, Lukkien et al’s [89] study emphasized the need for user-centered AI and the importance of contextual sensitivity in older adult care applications—themes that resonate with our focus on personalized interventions. Similar to Maugeri et al [14], we use both bibliometric and Google Trends data to analyze trends in health technologies. However, our study differs by incorporating content analysis, which offers deeper insights by examining the most-cited papers to explore study designs, popular topics, and potential limitations for future research.

Regarding EI, Zhou et al [10] explored EI’s potential to enhance real-time decision-making through edge computing, particularly in environments that generate vast amounts of data, such as those supported by IoT devices. Reis [90] emphasized EI’s role in optimizing operational efficiency in health care, particularly by processing data closer to the source to ensure timely care interventions. However, our study reveals that EI is not a standalone concept in LTC for older people. Instead, it functions as part of an integrated system, combining AI and IoT to support health care providers in making real-time decisions and offering personalized, technology-assisted interventions for care recipients. This fusion of technologies enables a more efficient and responsive older adult care environment, allowing health care providers to deliver tailored interventions based on immediate data, while providing older adults with customized assistive devices and services. By demonstrating how EI complements AI and IoT in this context, our study contributes to a deeper understanding of how these technologies work together to enhance care quality and personalization in LTC settings.

### Limitations

This study has several limitations. First, our analysis was confined to research from the past 10 years, even though the topic has been explored for over 3 decades. This choice was intentional to ensure the inclusion of the most recent and relevant data reflecting current trends. However, it may have excluded some foundational studies that provide historical context. Second, we only included English-language publications, which could introduce language bias and affect the global representativeness of our findings. Additionally, while content analysis was used to address some limitations of quantitative methods, it was restricted to the top 1% (n=64) of highly cited papers, potentially overlooking other important research. Future studies could benefit from adopting methods such as the Delphi study, as suggested by one of the reviewers, to gain deeper insights from expert opinions. This would further strengthen the qualitative depth of the research and make the study even more robust.

### Conclusions

The integration of AI, IoT, and EI is driving the evolution of an interconnected ecosystem in LTC for older adults. Our study underscores the significance of this field across multiple regions, emphasizing the need to promote diversity and international collaboration to enhance regional and institutional cooperation. Key themes such as “dementia care,” “machine learning,” and “wearable health monitoring” are emerging as focal areas of growing research interest. Keywords such as “long-term care,” “older adults,” “machine learning,” “dementia,” “smart home,” and “caregiver” demonstrate a strong correlation with the public interest, suggesting that incorporating these terms can increase research visibility. Despite the valuable insights into technology acceptance, care quality improvements, and outcomes from interventions, frequently cited studies often face limitations such as small sample sizes, short study durations, and a narrow focus on dementia care, restricting the generalizability of their findings. Future research should broaden the scope to include a wider range of health conditions, conduct long-term trials, and strengthen international cooperation. This approach will significantly enhance the scalability and real-world impact of these technologies globally.

## Appendix

Multimedia Appendix 1 Additional analysis and results.

## Abbreviations

AI: artificial intelligence
EI: edge intelligence
IoT: Internet of Things
LTC: long-term care
TGC: total global citation
TLC: total local citation

## Data Availability

All data generated or analyzed during this study are included in this published paper.
