# Supplementary material for: Use of Artificial Intelligence, Internet of Things, and Edge Intelligence in Long-Term Care for Older People: Comprehensive Analysis Through Bibliometric, Google Trends, and Content Analysis
Source: J Med Internet Res. 2025 Mar 4;27:e56692. doi: 10.2196/56692 (PMC11920668; doi:10.2196/56692)
Supplement: Multimedia Appendix 1 [file jmir_v27i1e56692_app1.docx]

Supplementary Materials

*Using Thematic Analysis for a Meticulous Examination Across Three Periods*

We conducted a thematic analysis, a qualitative method used to identify, analyze, and report patterns or themes within data, of author keywords across three distinct time periods: 2014–2016, 2017–2020, and 2021–2023, to capture the evolving research focus in AI, IoT, and EI applications for older adults in LTC [1]. As shown in **Figure S1**, the analysis maps the keywords based on two key dimensions: relevance (centrality), which measures how central a theme is to the overall research field, and development (density), which reflects how well-developed and internally coherent a theme is. The four quadrants in the figure represent different types of themes: Motor themes (high centrality, high density) are both highly relevant and well-developed; Niche themes (low centrality, high density) are specialized but less central; Basic themes (high centrality, low density) are foundational but underdeveloped; and Emerging or declining themes (low centrality, low density) are either nascent or losing prominence. This classification highlights shifting research priorities and emerging themes over time.


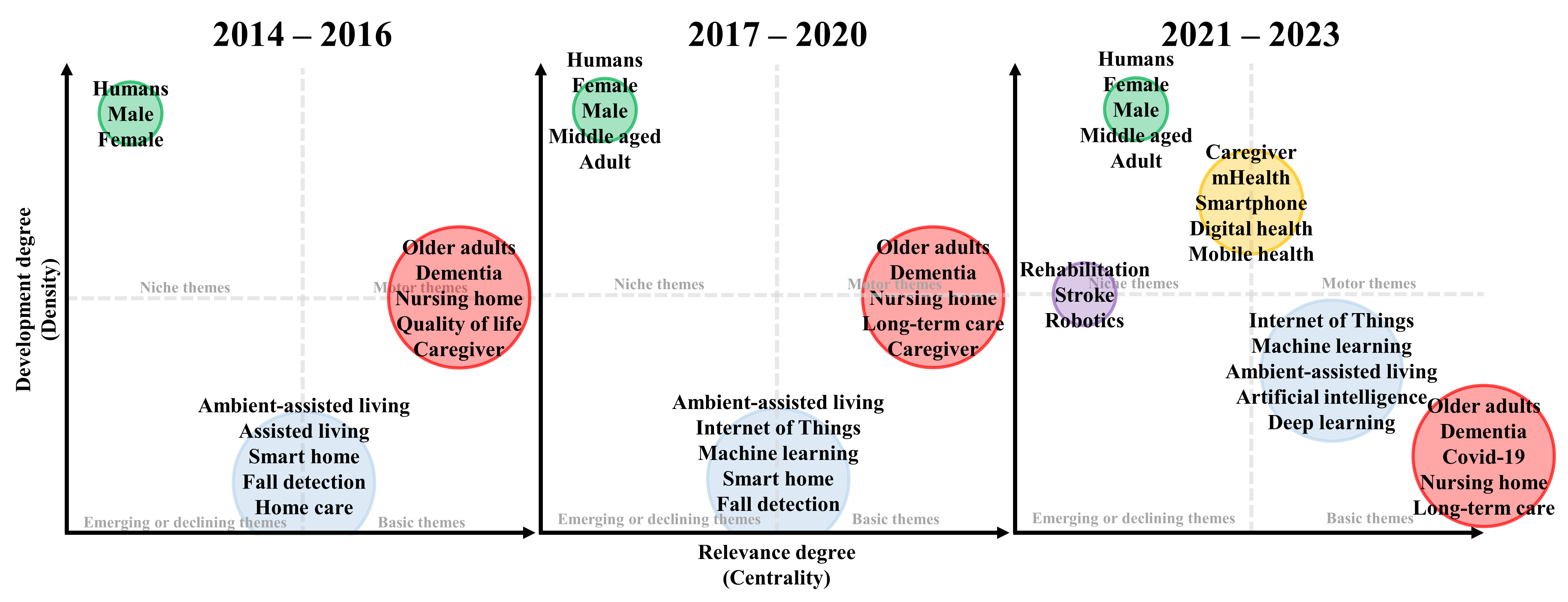


**Figure S1. Thematic evolution across three time periods (2014–2023).**

- **Phase I (2014–2016):** During this early period, the primary research focus centered around "older adults", "dementia", "nursing home", and "caregiver" as the dominant motor themes. Thematic clusters like "ambient-assisted living" and "fall detection" appeared as emerging or declining themes, indicating early interest in smart home technology and fall prevention. Meanwhile, "humans (male, female)" remained niche themes, reflecting more demographic-based research during this period.
- **Phase II (2017–2020):** The focus remained similar, with "older adults", "dementia", "nursing home", and "caregiver" continuing as core motor themes. However, new technologies such as "internet of things" and "machine learning" began to gain traction, shifting into the emerging or declining themes quadrant. The growing interest in these technologies signals a shift towards incorporating more advanced tech solutions, including smart home systems, ambient-assisted living, and fall detection.
- **Phase III (2021–2023):** The most recent period demonstrates a significant shift in research priorities. "older adults", "dementia", "long-term care", and the impact of Covid-19 have become highly central motor themes, reflecting the pandemic's profound effect on elder care research. Additionally, there is a notable increase in interest in digital and mobile health technologies, as keywords such as "mHealth", "digital health", and "smartphone" emerge as new key areas. Technologies like internet of things, artificial intelligence, deep learning, and rehabilitation also gained relevance, indicating a trend towards integrating more sophisticated AI-driven healthcare solutions.

Overall, the thematic analysis shows notable shifts in research priorities. "older adults", "dementia", and "nursing home" have consistently been central motor themes, but in the latest period (2021–2023), these themes expanded to include "long-term care" and "covid-19", reflecting the pandemic's significant impact on elder care research. New themes like "mHealth", "digital health", and "smartphone" emerged prominently in this period, highlighting the increasing focus on mobile and digital health solutions. Meanwhile, advanced technologies such as "artificial intelligence" and "deep learning" also gained relevance, indicating a growing trend towards more sophisticated AI applications in elder care. In contrast, themes like "fall detection" and "ambient-assisted living" remained less central, indicating their sustained but limited focus within the research landscape.

*Top 16 Frequent Keywords From Bibliometric Analysis and Their Google Trends Search Interest*

Through the bibliometric analysis of research from 2014 to 2023, we identified the top 16 most frequently occurring keywords in the context of AI, IoT, and EI applications for older adults in LTC. These keywords were categorized into two main groups: "older adults (OA)" and "ambient-assisted living (AAL)", based on the co-occurrence network and thematic analysis. We further utilized these keywords in Google Trends to supplement academic insights with public interest to assess societal relevance. Each keyword was expanded with four synonyms to ensure a comprehensive search scope. The related information, including category, keyword frequency, and corresponding synonyms, is detailed in **Table S1**.

**Table S1. Category, frequency, and synonyms for Google Trends search of top 16 keywords.**

| **#** | **Category** | **Original keywords** | **Frequency** | **Supplementary keywords for Google Trends searching:** |
| --- | --- | --- | --- | --- |
| **1** | OA | Older adults | 733 | Seniors, Elderly population, Aging adults, Older individuals |
| **2** | AAL | Ambient-assisted living | 404 | Smart living environments, Technology-assisted living, Intelligent care systems, Assisted home environments |
| **3** | OA | Dementia | 372 | Cognitive decline, Memory loss conditions, Neurodegenerative diseases, Alzheimer's disease |
| **4** | AAL | Internet of things | 353 | IoT devices, Connected devices, Smart devices network, Internet-connected systems |
| **5** | AAL | Machine learning | 287 | ML algorithms, Predictive models, Data-driven learning, AI learning systems |
| **6** | AAL | Smart home | 190 | Intelligent home systems, Connected home technology, Home automation, Smart residential systems |
| **7** | OA | Nursing home | 182 | Elderly care facility, Residential care home, Long-term care facility, Senior living center |
| **8** | OA | Caregiver | 174 | Care provider, Elderly support person, Home care assistant, Health care aide |
| **9** | AAL | Fall detection | 152 | Fall prevention systems, Fall monitoring, Accidental fall detection, Fall alert systems |
| **10** | AAL | Older adults care | 140 | Elderly care services, Senior care support, Aging population care, Care services for seniors |
| **11** | OA | Long-term care | 136 | Extended care services, Chronic care management, Ongoing health support, Continuous care services |
| **12** | OA | Quality of life | 134 | Life satisfaction, Well-being, Daily living quality, Personal health status |
| **13** | AAL | Assisted living | 126 | Supported living services, Assisted care facilities, Residential assisted care, Personal care homes |
| **14** | OA | Home care | 116 | In-home care services, Domiciliary care, At-home care support, Home-based care |
| **15** | AAL | Artificial intelligence | 111 | AI technology, Machine intelligence, Cognitive computing, Automated decision-making |
| **16** | OA | Aging | 108 | Getting older, Age-related changes, Senior aging process, Aging population dynamics |
| Abbreviation: OA = Older adults; AAL = Ambient-assisted living | | | | |

The "older adults" emerged as the most frequently used keyword, with 733 occurrences, followed by terms such as "ambient-assisted living" (n=404), "dementia" (n=372), and "internet of things" (n=353). These terms reflect the core focus areas in academic research, with a strong emphasis on addressing the healthcare needs of aging populations and exploring technological solutions to enhance care. Keywords such as "machine learning" (n=287), "artificial intelligence" (n=111), and "fall detection" (n=152) also highlight the growing interest in integrating advanced technologies into eldercare. The consistent rise in the occurrence of these keywords over the years, as depicted in the graph, indicates a sustained and increasing research focus on leveraging innovative technologies to improve the quality of life and care for older adults.

**Figure S2** shows the annual keyword occurrences for the top 16 keywords from 2014 to 2023, revealing notable shifts in frequency over the years. For example, "machine learning" first appeared in 2015 with 10 occurrences and steadily grew to 67 occurrences by 2023. Similarly, "internet of things" began with just one occurrence in 2014, but experienced rapid growth, reaching 49 occurrences in 2023. Keywords like "dementia" saw a significant rise from 19 occurrences in 2014 to a peak of 68 occurrences in 2020, before settling at 46 occurrences in 2023. Additionally, terms such as "caregiver" and "long-term care" showed moderate but consistent increases, particularly between 2016 and 2023, reflecting the growing academic interest in caregiving and extended care. On the other hand, while "ambient-assisted living" peaked at 59 occurrences in 2017, it gradually declined to 27 occurrences in 2023, suggesting a potential shift in focus toward other technological applications like IoT and AI.

**Figure S2. Annual increment of the top 16 common keywords.**

Next, we utilized the top 16 frequently occurring keywords identified from the bibliometric analysis, searching each in Google Trends alongside four additional synonyms to broaden the scope. The search was conducted globally, covering the period from January 1, 2014 to December 31, 2023. Google Trends provides search results on a scale of 0 to 100, where 100 represents the peak popularity of a keyword during the specified time, while 0 indicates insufficient data for that period [2]. To present the results visually, we plotted the search interest for each keyword. The larger dots in the figures represent the weighted average of the search interest, incorporating the keyword and its associated synonyms. The results are divided into two figures, grouped by category (OA and AAL), to illustrate the trends across different research areas and their corresponding public interest.

**Figure S3** illustrates the trends in public interest for keywords related to OA category from 2014 to 2023 based on Google Trends data. The figure highlights the growing search interest in terms such as "older adults", which consistently shows the highest search volume, peaking around 2023. Other related terms like "seniors", "elderly population", and "aging adults" follow a steady upward trend, although with lower search interest compared to "older adults". Similarly, terms like "caregiver", " long-term care", and "quality of life" show a gradual increase in search interest, reflecting public concerns and inquiries into eldercare. Overall, the public interest in topics related to older adults and LTC has seen moderate growth, as represented by the weighted average trends (red dots) for each keyword group, particularly in the latter half of the observed period.


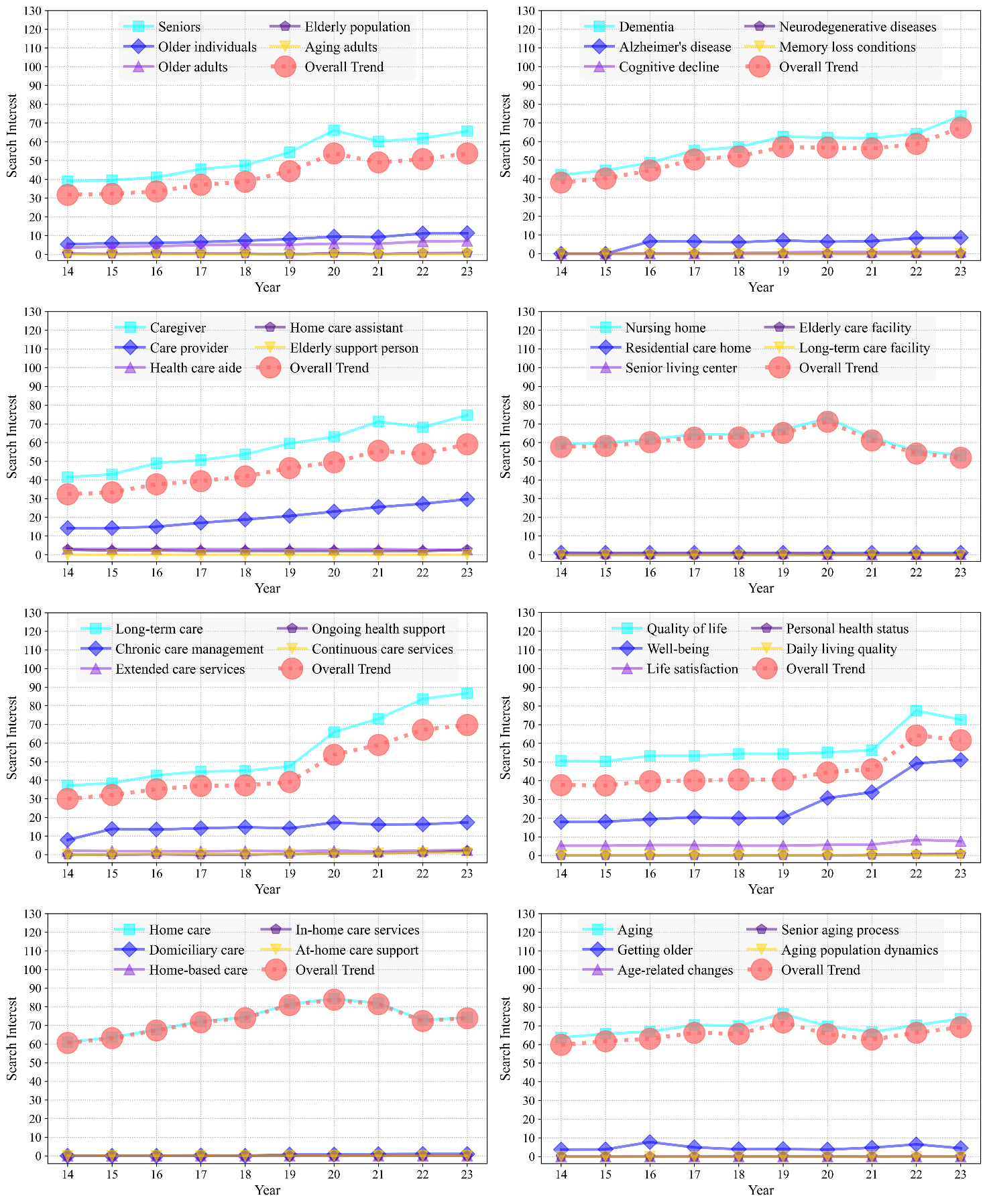


**Figure S3. Visualization of Google Trends (OA category) from 2014 to 2023.**

**Figure S4** displays the search interest trends for keywords related to AAL category from 2014 to 2023, based on Google Trends data. The figure shows that terms like "machine learning" and "artificial intelligence" saw significant increases in search interest, particularly from 2020 to 2023, reflecting growing public awareness and interest in the application of advanced technologies in assisted living. In contrast, the term "ambient-assisted living" experienced a decline in search interest during this period. Meanwhile, terms like "fall detection" maintained relatively low levels of search interest throughout the observed period. Overall, the weighted average trend (green dots) highlights an upward trajectory for technology-related topics within the AAL category, signaling increased public engagement with emerging technological solutions for eldercare.

**
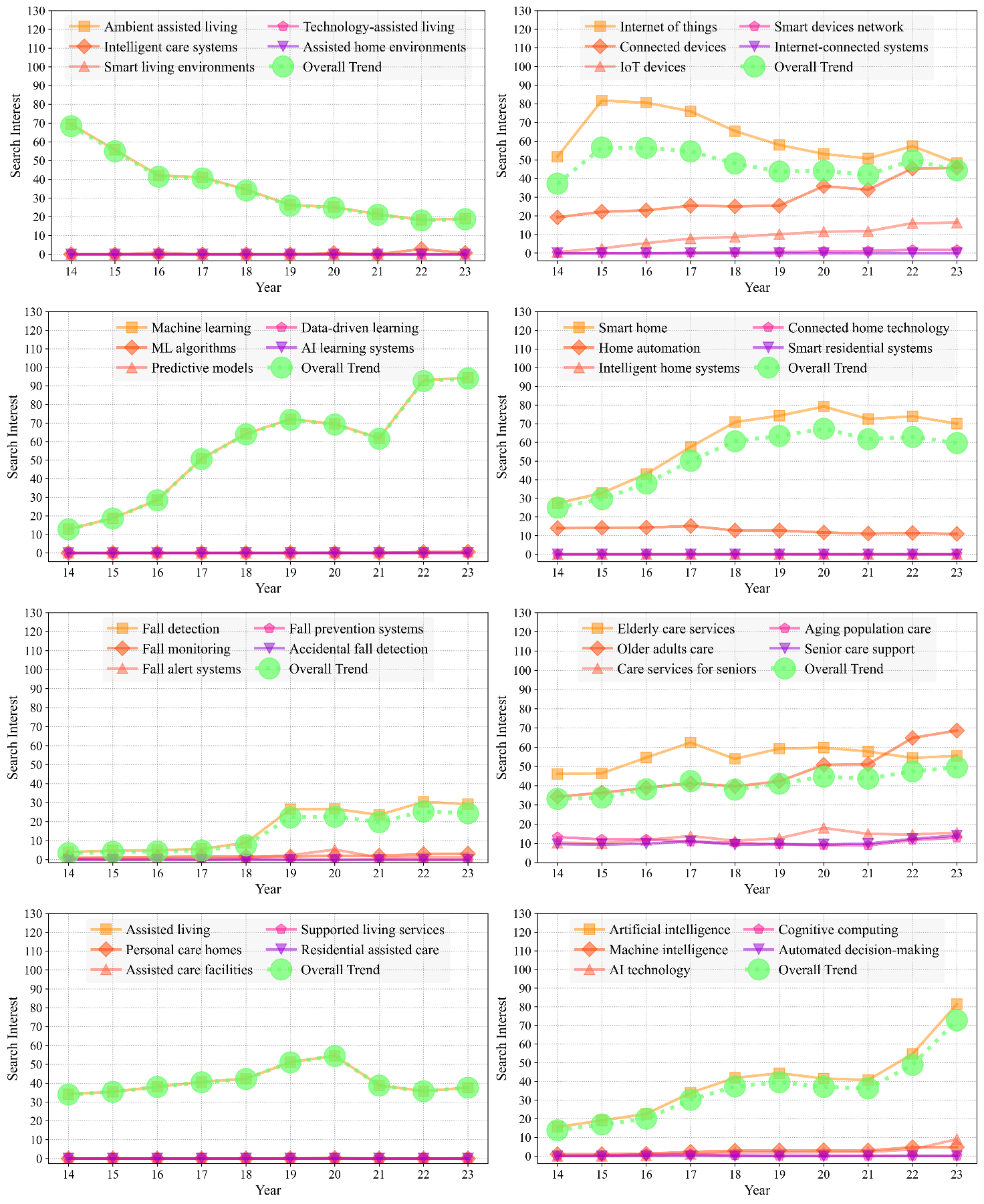
**

**Figure S4. Visualization of Google Trends (AAL category) from 2014 to 2023.**

Supplementary figures


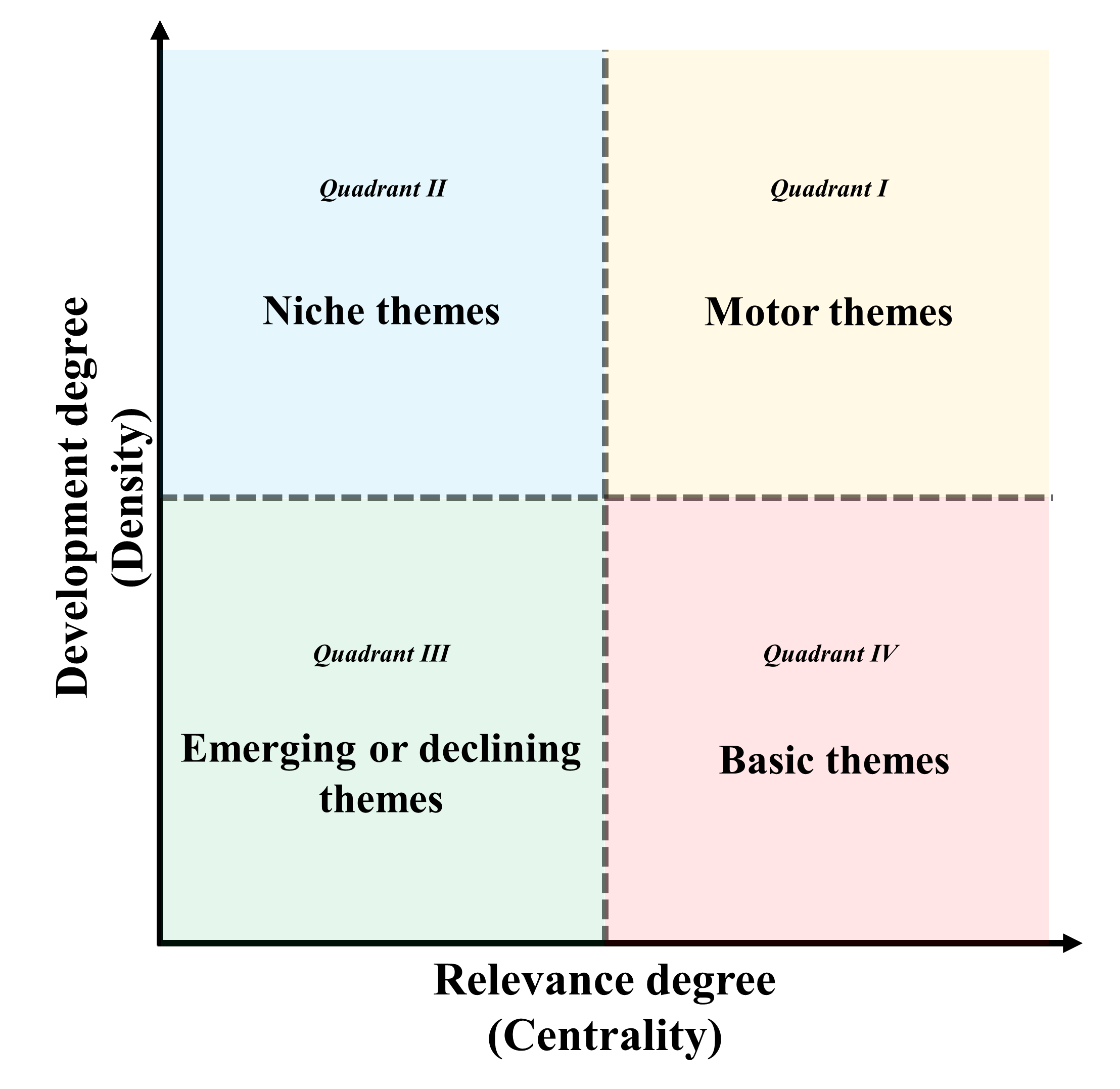


**Figure S5. Thematic map of research themes.**


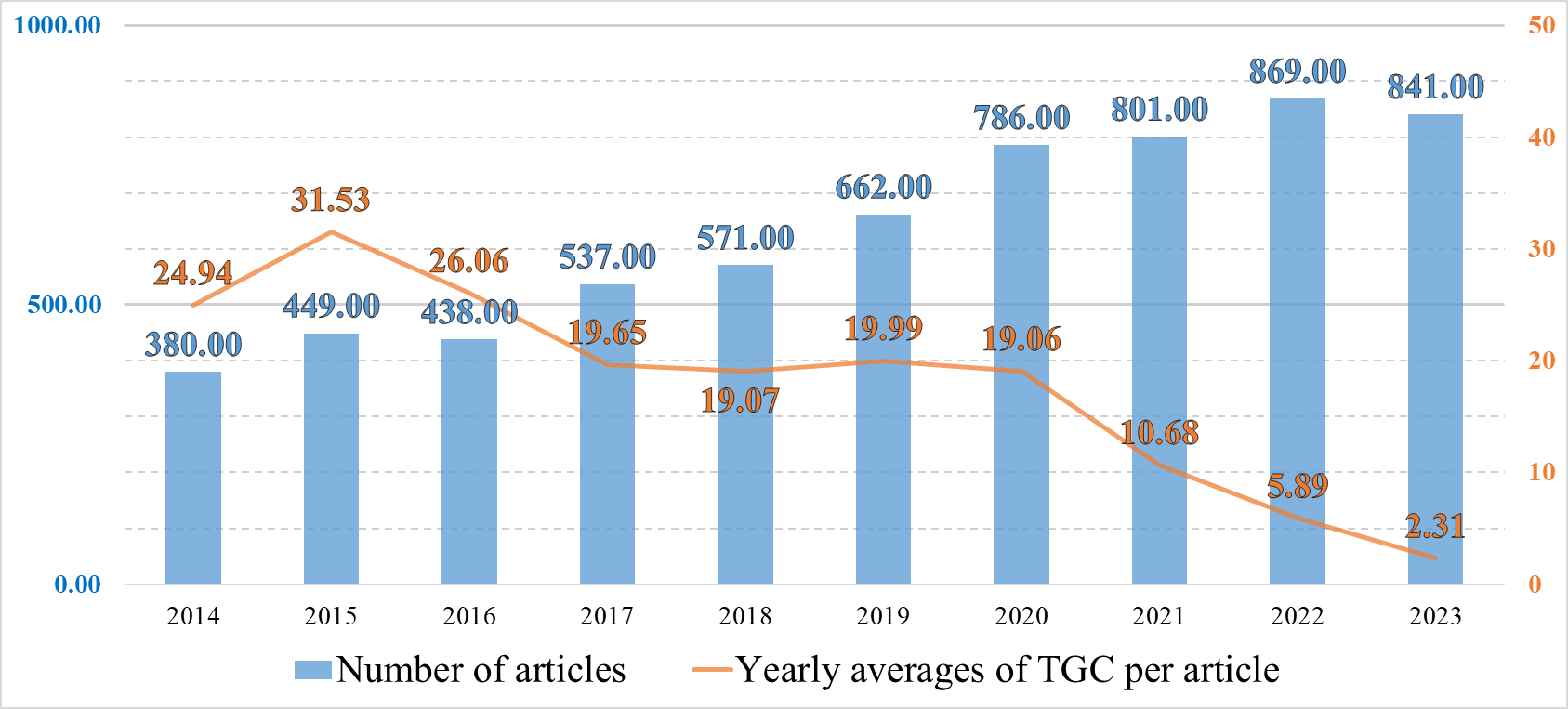


**Figure S6. Distribution of yearly publications and average TGC per article each year.**

Supplementary tables

**Table S2. Keywords for searching.**

| **#** | **Category** | **Keywords** |
| --- | --- | --- |
| **1** | Terms about older adults | ("older adult*" OR "elderly" OR "older person*" OR "senior*" OR "aged") |
| **2** | Terms linked to artificial intelligence (AI), the internet of things (IoT), or edge intelligence (EI) | ("artificial intelligence" OR "machine learning" OR "support vector machine" OR "neural networks" OR "vector machine" OR "machine learning" OR "deep learning" OR "ensemble learning" OR "deep network" OR "convolutional network" OR "neural network" OR "bayesian network" OR "classification tree" OR "regression tree" OR "probability tree" OR "nearest neighbor" OR "nearest neighbor" OR "fuzzy logit" OR "fuzzy logic" OR "fuzzy logistic" OR "native bayes" OR "genetic algorithm*" OR "multilayer perception" OR "random forest" OR "lasso*" OR "kernel*" OR "elastic net*" OR "fuzzy" OR “classification algorithm” OR “artificial neural network” OR "automated" OR "algorithm" OR "wearable*" OR "sensor*" OR "internet of things" OR "robot*" OR "digital human*" OR "virtual agent*" OR "conversation* agent" OR "chatbot" OR "app" OR "edge intelligence" OR "edge computing" OR "edge AI" OR "distributed AI" OR "fog computing" OR "cloudlet" OR "edge analytics" OR "edge processing" OR "intelligent edge" OR "edge cloud" OR "real-time analytics" OR "low-latency processing" OR "decentralized AI" OR "edge device*") |
| **3** | Terms associated with long-term care (LTC) | ("nursing home" OR "care home" OR "residential home" OR "extended care" OR "longterm care" OR "long-term care" OR "assisted living" OR "residential care" OR "caregiver" OR "social care" OR "home care" OR "home-based care" OR "respite care") |

**Table S3. Final search strategy used in each database.**

| **Database** | **Search Strategy** |
| --- | --- |
| **Web of Science** | TS=("older adult*" OR "elderly" OR "older person*" OR "senior*" OR "aged") AND TS=("artificial intelligence" OR "machine learning" OR "support vector machine" OR "neural networks" OR "vector machine" OR "machine learning" OR "deep learning" OR "ensemble learning" OR "deep network" OR "convolutional network" OR "neural network" OR "bayesian network" OR "classification tree" OR "regression tree" OR "probability tree" OR "nearest neighbor" OR "nearest neighbor" OR "fuzzy logit" OR "fuzzy logic" OR "fuzzy logistic" OR "native bayes" OR "genetic algorithm*" OR "multilayer perception" OR "random forest" OR "lasso*" OR "kernel*" OR "elastic net*" OR "fuzzy" OR “classification algorithm” OR “artificial neural network” OR "automated" OR "algorithm" OR "wearable*" OR "sensor*" OR "internet of things" OR "robot*" OR "digital human*" OR "virtual agent*" OR "conversation* agent" OR "chatbot" OR "app" OR "edge intelligence" OR "edge computing" OR "edge AI" OR "distributed AI" OR "fog computing" OR "cloudlet" OR "edge analytics" OR "edge processing" OR "intelligent edge" OR "edge cloud" OR "real-time analytics" OR "low-latency processing" OR "decentralized AI" OR "edge device*") AND TS=("nursing home" OR "care home" OR "residential home" OR "extended care" OR "longterm care" OR "long-term care" OR "assisted living" OR "residential care" OR "caregiver" OR "social care" OR "home care" OR "home-based care" OR "respite care") Timespan: 2014–2023; Language: English; Document Types: Article |
| **PubMed** | (("older adult*" OR "elderly" OR "older person*" OR "senior*" OR "aged") AND ("artificial intelligence" OR "machine learning" OR "support vector machine" OR "neural networks" OR "vector machine" OR "machine learning" OR "deep learning" OR "ensemble learning" OR "deep network" OR "convolutional network" OR "neural network" OR "bayesian network" OR "classification tree" OR "regression tree" OR "probability tree" OR "nearest neighbor" OR "nearest neighbor" OR "fuzzy logit" OR "fuzzy logic" OR "fuzzy logistic" OR "native bayes" OR "genetic algorithm*" OR "multilayer perception" OR "random forest" OR "lasso*" OR "kernel*" OR "elastic net*" OR "fuzzy" OR “classification algorithm” OR “artificial neural network” OR "automated" OR "algorithm" OR "wearable*" OR "sensor*" OR "internet of things" OR "robot*" OR "digital human*" OR "virtual agent*" OR "conversation* agent" OR "chatbot" OR "app" OR "edge intelligence" OR "edge computing" OR "edge AI" OR "distributed AI" OR "fog computing" OR "cloudlet" OR "edge analytics" OR "edge processing" OR "intelligent edge" OR "edge cloud" OR "real-time analytics" OR "low-latency processing" OR "decentralized AI" OR "edge device*") AND ("nursing home" OR "care home" OR "residential home" OR "extended care" OR "longterm care" OR "long-term care" OR "assisted living" OR "residential care" OR "caregiver" OR "social care" OR "home care" OR "home-based care" OR "respite care")) Filters: Publication dates: 2014–2023; Language: English; Article types: Journal articles only |
| **Scopus** | TITLE-ABS-KEY(("older adult*" OR "elderly" OR "older person*" OR "senior*" OR "aged") AND ("artificial intelligence" OR "machine learning" OR "support vector machine" OR "neural networks" OR "vector machine" OR "machine learning" OR "deep learning" OR "ensemble learning" OR "deep network" OR "convolutional network" OR "neural network" OR "bayesian network" OR "classification tree" OR "regression tree" OR "probability tree" OR "nearest neighbor" OR "nearest neighbor" OR "fuzzy logit" OR "fuzzy logic" OR "fuzzy logistic" OR "native bayes" OR "genetic algorithm*" OR "multilayer perception" OR "random forest" OR "lasso*" OR "kernel*" OR "elastic net*" OR "fuzzy" OR “classification algorithm” OR “artificial neural network” OR "automated" OR "algorithm" OR "wearable*" OR "sensor*" OR "internet of things" OR "robot*" OR "digital human*" OR "virtual agent*" OR "conversation* agent" OR "chatbot" OR "app" OR "edge intelligence" OR "edge computing" OR "edge AI" OR "distributed AI" OR "fog computing" OR "cloudlet" OR "edge analytics" OR "edge processing" OR "intelligent edge" OR "edge cloud" OR "real-time analytics" OR "low-latency processing" OR "decentralized AI" OR "edge device*") AND ("nursing home" OR "care home" OR "residential home" OR "extended care" OR "longterm care" OR "long-term care" OR "assisted living" OR "residential care" OR "caregiver" OR "social care" OR "home care" OR "home-based care" OR "respite care")) Date range: 2014–2023; Language: English; Document Type: Article |

**Table S4. Assessment tables for the enhanced** **mixed methods appraisal tool (MMAT).**

| **Category of study designs** | **Methodological quality criteria** | | **Responses** | | | |
| --- | --- | --- | --- | --- | --- | --- |
|  |  |  | Yes | No | Can’t tell | Comments |
| Screening questions (for all types) | S1. | Are there clear research questions? |  |  |  |  |
|  | S2. | Do the collected data allow to address the research questions? |  |  |  |  |
|  | *Further appraisal may not be feasible or appropriate when the answer is ‘No’ or ‘Can’t tell’ to one or both screening questions.* | | | | | |
| 1. Qualitative | 1.1. | Is the qualitative approach appropriate to answer the research question? |  |  |  |  |
|  | 1.2. | Are the qualitative data collection methods adequate to address the research question? |  |  |  |  |
|  | 1.3. | Are the findings adequately derived from the data? |  |  |  |  |
|  | 1.4. | Is the interpretation of results sufficiently substantiated by data? |  |  |  |  |
|  | 1.5. | Is there coherence between qualitative data sources, collection, analysis and interpretation? |  |  |  |  |
| 2. Quantitative randomized controlled trials | 2.1. | Is randomization appropriately performed? |  |  |  |  |
|  | 2.2. | Are the groups comparable at baseline? |  |  |  |  |
|  | 2.3. | Are there complete outcome data? |  |  |  |  |
|  | 2.4. | Are outcome assessors blinded to the intervention provided? |  |  |  |  |
|  | 2.5. | Did the participants adhere to the assigned intervention? |  |  |  |  |
| 3. Quantitative non-randomized | 3.1. | Are the participants representative of the target population? |  |  |  |  |
|  | 3.2. | Are measurements appropriate regarding both the outcome and intervention (or exposure)? |  |  |  |  |
|  | 3.3. | Are there complete outcome data? |  |  |  |  |
|  | 3.4. | Are the confounders accounted for in the design and analysis? |  |  |  |  |
|  | 3.5. | During the study period, is the intervention administered (or exposure occurred) as intended? |  |  |  |  |
| 4. Quantitative descriptive | 4.1. | Is the sampling strategy relevant to address the research question? |  |  |  |  |
|  | 4.2. | Is the sample representative of the target population? |  |  |  |  |
|  | 4.3. | Are the measurements appropriate? |  |  |  |  |
|  | 4.4. | Is the risk of nonresponse bias low? |  |  |  |  |
|  | 4.5. | Is the statistical analysis appropriate to answer the research question? |  |  |  |  |
| 5. Mixed methods | 5.1. | Is there an adequate rationale for using a mixed methods design to address the research question? |  |  |  |  |
|  | 5.2. | Are the different components of the study effectively integrated to answer the research question? |  |  |  |  |
|  | 5.3. | Are the outputs of the integration of qualitative and quantitative components adequately interpreted? |  |  |  |  |
|  | 5.4. | Are divergences and inconsistencies between quantitative and qualitative results adequately addressed? |  |  |  |  |
|  | 5.5. | Do the different components of the study adhere to the quality criteria of each tradition of the methods involved? |  |  |  |  |

**Table S5. Most productive institutions (sorted by the number of publications).**

| **#** | **Institutions** | **N** | **Location** |
| --- | --- | --- | --- |
| **1** | University of Toronto | 246 | Toronto, ON, Canada |
| **2** | University of California | 169 | Various Locations, USA |
| **3** | University of Michigan | 136 | Ann Arbor, MI, USA |
| **4** | University of Washington | 120 | Seattle, WA, USA |
| **5** | Griffith University | 106 | Brisbane, QLD, Australia |
| **6** | University of Pittsburgh | 102 | Pittsburgh, PA, USA |
| **7** | University of Waterloo | 99 | Waterloo, ON, Canada |
| **8** | Duke University | 98 | Durham, NC, USA |
| **9** | Harvard Medical School | 89 | Boston, MA, USA |
| **10** | University Health Network | 79 | Toronto, ON, Canada |
| **11** | McMaster University | 65 | Hamilton, ON, Canada |
| **12** | King’s College London | 58 | London, UK |
| **13** | University of Ottawa | 51 | Ottawa, ON, Canada |
| **14** | The University of Tokyo | 47 | Bunkyo City, Tokyo, Japan |
| **15** | University of British Columbia | 47 | Vancouver, BC, Canada |
| **16** | University of Oxford | 47 | Oxford, UK |
| **17** | Northwestern University | 43 | Evanston, IL, USA |
| **18** | University of Pennsylvania | 43 | Philadelphia, PA, USA |
| **19** | Public Health England Colindale | 42 | London, UK |
| **20** | The University of Manchester | 41 | Manchester, UK |
| Abbreviation: N = Number of publications | | | |

**Table S6. The keywords correlation between bibliometric analysis and Google Trends.**

| **#** | **Keywords** | **Scaled value** | | | | | | | | | | |  | **Correlation** | | |
| --- | --- | --- | --- | --- | --- | --- | --- | --- | --- | --- | --- | --- | --- | --- | --- | --- |
|  |  | **Types** | **2014** | **2015** | **2016** | **2017** | **2018** | **2019** | **2020** | **2021** | **2022** | **2023** |  | **tau** | | **P-value** |
| **1** | Older adults | BBA | 30.7 | 48.2 | 35.1 | 43.9 | 49.1 | 73.7 | 84.2 | 79.8 | 98.2 | 100.0 |  | 0.87 | *** | 0.0001 |
|  |  | GT | 27.9 | 28.3 | 29.5 | 32.6 | 34.0 | 39.0 | 47.2 | 43.1 | 44.6 | 47.2 |  |  |  |  |
| **2** | Ambient-assisted living | BBA | 28.1 | 36.8 | 36.0 | 51.8 | 33.3 | 42.1 | 33.3 | 38.6 | 30.7 | 23.7 |  | 0.18 |  | 0.4725 |
|  |  | GT | 60.0 | 48.2 | 36.3 | 35.5 | 29.8 | 22.8 | 21.8 | 18.5 | 15.9 | 16.4 |  |  |  |  |
| **3** | Dementia | BBA | 16.7 | 20.2 | 20.2 | 22.8 | 22.8 | 34.2 | 59.6 | 35.1 | 54.4 | 40.4 |  | 0.75 | ** | 0.0029 |
|  |  | GT | 33.4 | 35.3 | 39.0 | 44.2 | 45.7 | 50.1 | 49.7 | 49.3 | 51.5 | 59.1 |  |  |  |  |
| **4** | Internet of things | BBA | 0.9 | 6.1 | 13.2 | 33.3 | 26.3 | 39.5 | 46.5 | 46.5 | 54.4 | 43.0 |  | -0.22 |  | 0.3692 |
|  |  | GT | 32.6 | 49.7 | 49.5 | 47.8 | 42.2 | 38.4 | 38.6 | 36.9 | 43.7 | 38.9 |  |  |  |  |
| **5** | Machine learning | BBA | 0.0 | 8.8 | 7.9 | 13.2 | 13.2 | 23.7 | 40.4 | 39.5 | 46.5 | 58.8 |  | 0.81 | ** | 0.0012 |
|  |  | GT | 11.2 | 16.3 | 24.9 | 44.4 | 56.1 | 63.0 | 60.7 | 54.0 | 81.2 | 82.5 |  |  |  |  |
| **6** | Smart home | BBA | 9.6 | 13.2 | 13.2 | 17.5 | 18.4 | 21.9 | 24.6 | 16.7 | 16.7 | 14.9 |  | 0.75 | ** | 0.0029 |
|  |  | GT | 21.9 | 26.0 | 33.3 | 44.0 | 53.2 | 55.6 | 59.0 | 54.1 | 55.2 | 52.3 |  |  |  |  |
| **7** | Nursing home | BBA | 9.6 | 10.5 | 12.3 | 8.8 | 10.5 | 14.0 | 21.1 | 24.6 | 25.4 | 22.8 |  | -0.04 |  | 0.8575 |
|  |  | GT | 50.6 | 51.1 | 52.8 | 54.9 | 55.1 | 57.1 | 62.4 | 53.6 | 47.5 | 45.5 |  |  |  |  |
| **8** | Caregiver | BBA | 5.3 | 8.8 | 7.9 | 5.3 | 12.3 | 11.4 | 20.2 | 19.3 | 30.7 | 31.6 |  | 0.72 | ** | 0.0041 |
|  |  | GT | 28.4 | 29.3 | 33.1 | 34.5 | 36.7 | 40.7 | 43.4 | 48.8 | 47.3 | 51.8 |  |  |  |  |
| **9** | Fall detection | BBA | 7.9 | 12.3 | 11.4 | 8.8 | 11.4 | 9.6 | 15.8 | 21.9 | 14.9 | 19.3 |  | 0.40 |  | 0.1060 |
|  |  | GT | 3.0 | 3.6 | 3.8 | 4.3 | 6.7 | 19.5 | 19.9 | 17.3 | 22.3 | 21.5 |  |  |  |  |
| **10** | Older adults care | BBA | 4.4 | 4.4 | 10.5 | 11.4 | 12.3 | 7.0 | 15.8 | 17.5 | 20.2 | 19.3 |  | 0.72 | ** | 0.0041 |
|  |  | GT | 29.0 | 29.7 | 33.5 | 37.2 | 33.3 | 36.0 | 39.2 | 38.3 | 41.6 | 43.4 |  |  |  |  |
| **11** | Long-term care | BBA | 2.6 | 5.3 | 5.3 | 8.8 | 11.4 | 15.8 | 16.7 | 14.9 | 16.7 | 21.9 |  | 0.89 | *** | 0.0004 |
|  |  | GT | 26.3 | 28.1 | 30.9 | 32.4 | 32.7 | 34.2 | 47.1 | 51.7 | 58.9 | 61.1 |  |  |  |  |
| **12** | Quality of life | BBA | 8.8 | 7.9 | 7.0 | 6.1 | 6.1 | 18.4 | 15.8 | 14.9 | 12.3 | 20.2 |  | 0.31 |  | 0.2087 |
|  |  | GT | 33.0 | 32.9 | 34.9 | 35.2 | 35.6 | 35.7 | 38.9 | 40.4 | 56.3 | 54.1 |  |  |  |  |
| **13** | Assisted living | BBA | 6.1 | 14.9 | 16.7 | 7.0 | 9.6 | 11.4 | 15.8 | 13.2 | 6.1 | 9.6 |  | 0.30 |  | 0.2411 |
|  |  | GT | 29.8 | 31.0 | 33.3 | 35.5 | 37.1 | 44.8 | 47.7 | 34.0 | 31.3 | 33.0 |  |  |  |  |
| **14** | Home care | BBA | 6.1 | 11.4 | 5.3 | 4.4 | 10.5 | 7.9 | 10.5 | 12.3 | 17.5 | 15.8 |  | 0.13 |  | 0.5900 |
|  |  | GT | 53.2 | 55.4 | 59.1 | 62.9 | 64.9 | 71.0 | 73.5 | 71.4 | 63.5 | 64.8 |  |  |  |  |
| **15** | Artificial intelligence | BBA | 0.0 | 1.8 | 0.0 | 1.8 | 1.8 | 7.0 | 15.8 | 22.8 | 15.8 | 30.7 |  | 0.61 | * | 0.0173 |
|  |  | GT | 12.1 | 14.9 | 17.6 | 26.5 | 32.8 | 34.7 | 32.5 | 31.9 | 42.9 | 63.8 |  |  |  |  |
| **16** | Aging | BBA | 2.6 | 8.8 | 7.9 | 4.4 | 10.5 | 5.3 | 13.2 | 7.9 | 18.4 | 15.8 |  | 0.27 |  | 0.2812 |
|  |  | GT | 52.5 | 54.2 | 55.3 | 58.1 | 57.6 | 62.9 | 57.5 | 55.0 | 58.1 | 60.8 |  |  |  |  |
| Abbreviation: BBA = Bibliometric analysis, GT = Google Trends | | | | | | | | | | | | | | | | |

**Table S7. Quality assessment outcomes from 64 content analysis studies.**

| **#** | **Articles** | **Category of**  **study designs** | **S1** | **S2** | **Ⅰ** | **Ⅱ** | **Ⅲ** | **Ⅳ** | **Ⅴ** | **Explanation** |
| --- | --- | --- | --- | --- | --- | --- | --- | --- | --- | --- |
| **1** | Joranson N et al., 2015 [3] | Quantitative randomized controlled trials | Yes | Yes | Yes | Yes | Yes | No^1^ | Yes | 1. Blinding the assessors or participants was not possible due to the nature of the intervention, as acknowledged by the authors. |
| **2** | Moyle W et al., 2017 [4] | Quantitative randomized controlled trials | Yes | Yes | Yes | Yes | No^1^ | Yes | Yes | 1. Some participants were lost to follow-up due to death, refusal, or unavailability, and the data for these participants were handled using imputation methods |
| **3** | Pino M et al., 2015 [5] | Mixed methods | Yes | Yes | Yes | Yes | Yes | Can't Tell^1^ | Yes | 1. The paper does not explicitly mention whether inconsistencies were found between the two types of data or how they were addressed, so this point remains unclear. |
| **4** | Moyle W et al., 2018 [6] | Qualitative | Yes | Yes | Yes | Yes | Yes | Yes | Yes |  |
| **5** | Liang A et al., 2017 [7] | Quantitative randomized controlled trials | Yes | Yes | Yes | Yes | No^1^ | Yes | Yes | 1. Some participants dropped out due to health deterioration, resulting in incomplete data for certain measures such as hair cortisol |
| **6** | Joranson N et al., 2016 [8] | Quantitative randomized controlled trials | Yes | Yes | Yes | Yes | No^1^ | No^2^ | Yes | 1. Some participants were lost to follow-up due to mortality and other reasons. Although the analysis accounted for missing data using multiple imputation, the outcome data is not fully complete. 2. Blinding of assessors was not possible in this type of intervention study, which the authors acknowledge as a limitation |
| **7** | Thodberg K et al., 2016 [9] | Quantitative randomized controlled trials | Yes | Yes | Yes | Yes | Yes | Can't Tell^1^ | Yes | 1. The description of blinding in outcome assessment is not explicitly mentioned. |
| **8** | Moyle W et al., 2014 [10] | Mixed methods | Yes | Yes | Yes | Yes | Yes | Can't Tell^1^ | Yes | 1. There is no explicit mention of addressing potential inconsistencies between the qualitative and quantitative components. |
| **9** | Moyle W et al., 2015 [11] | Quantitative randomized controlled trials | Yes | Yes | Yes | Yes | Yes | Yes | Yes |  |
| **10** | Robinson H et al., 2015 [12] | Quantitative non-randomized | Can't Tell^1^ | Yes | Yes | Yes | Yes | No^2^ | Yes | 1. Limited information on how participants were selected or their general representativeness. 2. There is no detailed mention of adjustments for potential confounding variables. |
| **11** | Gustafsson C et al., 2015 [13] | Mixed methods | Yes | Yes | Yes | Yes | Yes | Can't Tell^1^ | Yes | 1. No clear mention of how inconsistencies between qualitative and quantitative data were managed. |
| **12** | Yamada Y et al., 2014 [14] | Quantitative descriptive | Yes | Can't Tell^1^ | Yes | Yes | Yes | Can't Tell^2^ | Yes | 1. The study does not specify if the nursing homes were randomly selected, which limits the generalizability. 2. There is no explicit mention of response rates or how nonresponses were handled. |
| **13** | Moyle W et al., 2019 [15] | Mixed methods | Yes | Yes | Yes | Yes | Yes | Can't Tell^1^ | Yes | 1. There is no specific mention of handling inconsistencies between qualitative and quantitative results. |
| **14** | Moyle W et al., 2019 [16] | Qualitative | Yes | Yes | Yes | Yes | Yes | Yes | Yes |  |
| **15** | Moyle W et al., 2016 [17] | Mixed methods | Yes | Yes | Yes | Yes | Yes | Can't Tell^1^ | Yes | 1. The paper does not explicitly mention how inconsistencies between qualitative and quantitative findings are handled. |
| **16** | Koceski S et al., 2016 [18] | Quantitative descriptive | Yes | Yes | Yes | No^1^ | Yes | Can't Tell^2^ | Yes | 1. The study included only 35 participants from a single nursing home, which may not fully represent the broader elderly population or all caregiving environments. 2. The study does not provide detailed information on participant dropout rates or the extent to which all participants completed the questionnaires. |
| **17** | Mitoku K et al., 2016 [19] | Quantitative descriptive | Yes | Yes | Yes | No^1^ | Yes | Can't Tell^2^ | Yes | 1. The study is limited to LTC recipients in a rural area of Japan, which may not fully represent the broader elderly population or urban settings. 2. The study does not provide detailed information on participant dropout rates or potential biases from nonresponse. |
| **18** | Thodberg K et al., 2016 [20] | Quantitative randomized controlled trials | Yes | Yes | Yes | Can't Tell^1^ | No^2^ | Can't Tell^3^ | Yes | 1. The baseline characteristics are not fully described in terms of comparability between groups beyond a few demographic details. 2. 23 participants dropped out during the experimental period, but it’s unclear how this dropout affected the final analysis. 3. It is not specified whether outcome assessors were blinded to the intervention. |
| **19** | Bemelmans R et al., 2015 [21] | Quantitative non-randomized | Yes | Yes | Yes | Yes | No^1^ | No^2^ | Yes | 1. Some participants (20) dropped out during the study, resulting in incomplete data. 2. The study does not mention adjusting for confounding variables, such as the stage of dementia or variability in care staff involvement. |
| **20** | Claes V et al., 2015 [22] | Quantitative descriptive | Yes | Yes | Yes | No^1^ | Yes | Can't Tell^2^ | Yes | 1. The sample consists of members from specific patient and consumer organizations, which may not fully represent the broader population of older adults. 2. The study does not provide detailed information on the response rate or potential nonresponse bias. |
| **21** | Broadbent E et al., 2016 [23] | Quantitative non-randomized | Yes | Yes | Yes | Yes | No^1^ | No^2^ | Yes | 1. Some participants did not complete the follow-up, leading to missing data. 2. The study does not mention adjusting for potential confounders such as variations in cognitive ability or pre-existing conditions among residents. |
| **22** | Rantanen T et al., 2018 [24] | Quantitative descriptive | Yes | Yes | Yes | No^1^ | Yes | No^2^ | Yes | 1. The response rate was relatively low (18.2%), and the sample may be biased towards those with more favorable attitudes towards care robots. 2. The response rate was low, and it is possible that non-respondents had different attitudes, which could introduce bias. |
| **23** | Tang V et al., 2019 [25] | Quantitative descriptive | Yes | Yes | Yes | No^1^ | Yes | Can't Tell^2^ | Yes | 1. The sample is limited to one nursing home in Taiwan, which may not be fully representative of nursing homes in different regions or contexts. 2. The study does not provide detailed information on whether there were nonrespondents or how the data collection process might have been affected by participation rates. |
| **24** | Rantanen T et al., 2018 [26] | Quantitative descriptive | Yes | Yes | Yes | Yes | Yes | No^1^ | Yes | 1. The study reports a response rate of 18.2%, which raises concerns about nonresponse bias, although two municipalities achieved higher response rates. |
| **25** | Moyle W et al., 2018 [27] | Quantitative randomized controlled trials | Yes | Yes | Yes | Yes | No^1^ | Yes | Yes | 1. A significant number of participants were excluded due to not meeting the wear-time criteria for the accelerometers, which may impact the completeness of the data. |
| **26** | Shiomi M et al., 2015 [28] | Quantitative descriptive | Yes | Yes | Yes | Yes | Yes | Can't Tell^1^ | Yes | 1. While the study reports the number of participants, it is unclear whether all approached individuals participated or if there was any selection bias. |
| **27** | Diraco G et al., 2017 [29] | Quantitative descriptive | Yes | Yes | Yes | Can't Tell^1^ | Yes | Can't Tell^2^ | Yes | 1. The document mentions two age groups but does not specify whether they fully represent the elderly population, which is the intended focus of the sensor. 2. The study does not provide information on whether any participants dropped out or failed to complete the tasks, leaving uncertainty about the presence of nonresponse bias. |
| **28** | Korchut A et al., 2017 [30] | Qualitative | Yes | Yes | Yes | Yes | Yes | Yes | Yes |  |
| **29** | Berridge C, 2016 [31] | Qualitative | Yes | Yes | Yes | Yes | Yes | Yes | Yes |  |
| **30** | Berridge C, 2017 [32] | Qualitative | Yes | Yes | Yes | Yes | Yes | Yes | Yes |  |
| **31** | Machado A et al., 2017 [33] | Quantitative descriptive | Yes | Yes | Can't Tell^1^ | Can't Tell^2^ | Yes | Can't Tell^3^ | Yes | 1. The document does not provide detailed information about the sampling strategy or how participants or environments were selected. 2. There is no information on whether the sample used in the case study (simulated smart homes) represents the broader target population of ambient-assisted living environment. 3. The document does not mention nonresponse bias or provide details on how missing data (if any) was handled. |
| **32** | Erebak S et al., 2019 [34] | Quantitative non-randomized | Yes | Yes | Can't Tell^1^ | Yes | Can't Tell^2^ | Yes | Yes | 1. The study includes caregivers from a specific elder care facility, but the document does not mention how well the sample represents the broader population of caregivers working in elder care. 2. The study does not explicitly mention whether potential confounders, such as caregivers' prior experience with technology or robots, were accounted for in the design or analysis. |
| **33** | Jones C et al., 2018 [35] | Quantitative descriptive | Yes | Yes | Yes | Yes | Yes | Yes | Yes |  |
| **34** | Al-Khafajiy M et al., 2019 [36] | Quantitative descriptive | Yes | Yes | Can't Tell^1^ | Can't Tell^2^ | Yes | Can't Tell^3^ | Yes | 1. The document focuses more on system performance and technology rather than describing how participants or environments were sampled. 2. The study does not provide specific details on the demographic representativeness of the elderly participants using the system. 3. The document does not mention how missing data or nonresponse was handled, leaving this unclear. |
| **35** | Rafferty J et al., 2017 [37] | Quantitative descriptive | Yes | Yes | Can't Tell^1^ | Can't Tell^2^ | Yes | Can't Tell^3^ | Yes | 1. The document does not provide detailed information on the participant or environmental sampling strategy used to test the system in the smart home setting. 2. The study does not specify if the participants or testing environments used in the three scenarios are representative of the broader population of individuals who might use smart home technology. 3. There is no mention of nonresponse or dropout rates in the evaluation scenarios, making it unclear if nonresponse bias is a concern. |
| **36** | Lloret J et al., 2015 [38] | Quantitative descriptive | Yes | Yes | Can't Tell^1^ | Can't Tell^2^ | Yes | Can't Tell^3^ | Yes | 1. The study primarily focuses on system architecture and performance metrics, with limited information on the sampling strategy for real-world testing. 2. The study is more focused on technological validation, and does not provide specific details about participants or end users involved in testing. 3. The document does not discuss nonresponse or dropout, leaving this unclear. |
| **37** | Decerle J et al., 2019 [39] | Quantitative descriptive | Yes | Yes | Can't Tell^1^ | Can't Tell^2^ | Yes | Can't Tell^3^ | Yes | 1. The study relies on computational instances rather than real-world sampling, which is typical for algorithmic research, but the document does not clarify if the test instances reflect actual home health care conditions. 2. Since the study is based on computational simulations, it is not clear how representative the test instances are of real-world home health care scenarios. 3. There is no discussion of nonresponse bias, as the study uses computational simulations, so this question is not directly applicable. |
| **38** | Pfadenhauer M et al., 2015 [40] | Qualitative | Yes | Yes | Yes | Yes | Yes | Yes | Yes |  |
| **39** | Cavallo F et al., 2014 [41] | Mixed methods | Yes | Yes | Yes | Yes | Yes | Can't Tell^1^ | Yes | 1. The document does not explicitly discuss any inconsistencies between the qualitative and quantitative components of the study. |
| **40** | Nikoloudakis Y et al., 2016 [42] | Quantitative descriptive | Yes | Yes | Can't Tell^1^ | Can't Tell^2^ | Yes | Can't Tell^3^ | Yes | 1. The study focuses on system performance rather than participant sampling, and there is limited information on the sampling of test environments or users. 2. The research describes testing conditions but does not provide detailed information on whether the environments or participants are fully representative of the intended real-world user base. 3. The study does not mention any nonresponse or dropout rates, but since it relies on system performance metrics, the concept of nonresponse may not apply directly. |
| **41** | Atee M et al., 2017 [43] | Quantitative descriptive | Yes | Yes | Yes | Can't Tell^1^ | Yes | Yes | Yes | 1. The study sample includes mostly Caucasian females, which may not fully represent the broader population of dementia patients in diverse settings. |
| **42** | Pigini L et al., 2017 [44] | Mixed methods | Yes | Yes | Yes | Yes | Yes | Can't Tell^1^ | Yes | 1. The document does not specifically discuss inconsistencies between qualitative and quantitative data, making it unclear whether any were encountered or addressed. |
| **43** | Jones C et al., 2018 [45] | Quantitative randomized controlled trials | Yes | Yes | Yes | Yes | Yes | Can't Tell^1^ | Yes | 1. The document does not explicitly state whether the outcome assessors were blinded, though video observations were used, which suggests possible blinding. |
| **44** | Lehoux P et al., 2018 [46] | Qualitative | Yes | Yes | Yes | Yes | Yes | Yes | Yes |  |
| **45** | Mulero R et al., 2018 [47] | Quantitative descriptive | Yes | Yes | Yes | Yes | Yes | Can't Tell^1^ | Yes | 1. The document does not provide detailed information on nonresponse rates or how missing data from the elderly population was handled. |
| **46** | Darragh M et al., 2017 [48] | Qualitative | Yes | Yes | Yes | Yes | Yes | Yes | Yes |  |
| **47** | D'Onofrio G et al., 2019 [49] | Qualitative | Yes | Yes | Yes | Yes | Yes | Yes | Yes |  |
| **48** | Khosla R et al., 2021 [50] | Mixed methods | Yes | Yes | Yes | Yes | Yes | Can't Tell^1^ | Yes | 1. The document does not explicitly discuss whether there were any inconsistencies between the qualitative observations and the quantitative survey results. |
| **49** | Cortellessa G et al., 2018 [51] | Quantitative descriptive | Yes | Yes | Yes | Yes | Yes | Can't Tell^1^ | Yes | 1. The study does not provide detailed information on response rates or potential nonresponse bias. |
| **50** | Wright J, 2019 [52] | Qualitative | Yes | Yes | Yes | Yes | Yes | Yes | Yes |  |
| **51** | Lukasik S et al., 2020 [53] | Quantitative descriptive | Yes | Yes | Yes | Yes | Yes | Can't Tell^1^ | Yes | 1. The document does not provide detailed information about the nonresponse rate, so it's unclear whether nonresponse bias is a concern. |
| **52** | Pouyet V et al., 2015 [54] | Quantitative non-randomized | Yes | Yes | Yes | Yes | Yes | Can't Tell^1^ | Yes | 1. While the study considers cognitive status, it is unclear whether other potential confounders, such as physical health or appetite, were controlled for during analysis. |
| **53** | Wittich W et al., 2018 [55] | Qualitative | Yes | Yes | Yes | Yes | Yes | Yes | Yes |  |
| **54** | Yamada Y et al., 2015 [56] | Quantitative non-randomized | Yes | Yes | Yes | Yes | Yes | Yes | Yes |  |
| **55** | Daniele K et al., 2019 [57] | Qualitative | Yes | Yes | Yes | Yes | Yes | Yes | Yes |  |
| **56** | Yu R et al., 2014 [58] | Quantitative descriptive | Yes | Yes | Yes | Yes | Yes | Can't Tell^1^ | Yes | 1. There is no specific mention of nonresponse rates or how nonresponse might have been addressed. |
| **57** | Chetty G et al., 2015 [59] | Quantitative descriptive | Yes | Yes | Yes | Yes | Yes | Can't Tell^1^ | Yes | 1. The study uses a pre-collected dataset, and there is no information about nonresponse bias or how it might affect the results. |
| **58** | Arends J et al., 2018 [60] | Quantitative non-randomized | Yes | Yes | Yes | Yes | Yes | Can't Tell^1^ | Yes | 1. There is limited information on how confounders, such as participant movement disorders or medication changes, were addressed in the analysis. |
| **59** | Lazarou I et al., 2016 [61] | Quantitative non-randomized | Yes | Yes | Yes | Yes | Can't Tell^1^ | Can't Tell^2^ | Yes | 1. While there is extensive data collected, it is not clear whether all data points for all participants were complete throughout the study. 2. The document doesn't explicitly mention adjusting for confounders, such as variations in disease severity or other health conditions that could influence the outcomes. |
| **60** | Meritam P et al., 2018 [62] | Quantitative non-randomized | Yes | Yes | Yes | Yes | Yes | Can't Tell^1^ | Yes | 1. The study does not explicitly discuss controlling for confounding factors that could affect device performance, such as patient activity levels or other seizure types. |
| **61** | Schwenk M et al., 2014 [63] | Quantitative non-randomized | Yes | Yes | Yes | Yes | No^1^ | Can't Tell^2^ | Yes | 1. The study had a 25.2% dropout rate, and not all participants completed the fall documentation. 2. While previous falls are considered, it is unclear whether other potential confounders (e.g., comorbidities) were fully controlled for in the analysis. |
| **62** | Martinikorena I et al., 2016 [64] | Quantitative descriptive | Yes | Yes | Yes | Yes | Yes | Can't Tell^1^ | Yes | 1. The study does not mention if all eligible participants agreed to participate or how nonresponders might differ from responders. |
| **63** | Ho A, 2020 [65] | Qualitative | Yes | Yes | Yes | Yes | Yes | Yes | Yes |  |
| **64** | Lattanzio F et al., 2014 [66] | Qualitative | Yes | Yes | Yes | Yes | Yes | Yes | Yes |  |
| Abbreviation: LTC = long-term care | | | | | | | | | | |

**Table S8 Content analysis of top 1% impactful papers.**

| **#** | **Articles** | **C/R (CA)** | **S/M-center** | **N** | **Age** | **%F** | **ITP** | **Assistive robot** | **Objective** | **Findings** | **Limitations** |
| --- | --- | --- | --- | --- | --- | --- | --- | --- | --- | --- | --- |
| **1** | Joranson N et al., 2015 [3] | Norway | Multi | 60 | 62–95 | 67 | Dementia | PARO | - Examine the effects of robot-assisted activity on agitation and depression in persons with dementia. - Evaluate the use of PARO in nursing homes. - Assess the long-term impacts of such interventions. | - PARO reduces agitation and depression among dementia patients. - The effects are long-term, lasting even three months postintervention. - The psychosocial environment in nursing homes contributes to positive outcomes. | - Activity in the control group was not monitored. - Blinding was not possible due to the nature of the intervention. - Cluster design might have introduced environmental influences. |
| **2** | Moyle W et al., 2017 [4] | Australia | Multi | 415 | 60+ | 76 | Dementia | PARO | - Test the effects of PARO against plush toy and usual care on dementia symptoms. | - PARO led to greater engagement than plush toy. | - Short duration might have influenced results. |
| **3** | Pino M et al., 2015 [5] | France | Single | 25 | 58–86 | 68 | MCI, Mild cognitive impairment, dementia | RobuLAB 10 | - Investigate SAR acceptance among older adults. - Examine how opinions differ between healthy elderly, individuals with MCI, and informal caregivers. | - SAR can support cognitive and social needs. - SAR's appearance and usability affect acceptance. - Customization enhances acceptance. | - Small sample size limits generalizability. - Lack of direct interaction between participants and SAR. |
| **4** | Moyle W et al., 2018 [6] | Australia | Multi | 20 | 20–75 | 50 | Dementia | PARO | - Explore care staff perceptions of PARO and a look-alike non-robotic animal in dementia care. - Examine benefits and limitations of PARO compared to the plush toy. | - PARO improved mood, engagement, and reduced agitation in residents. - Plush Toy did not engage residents meaningfully. - PARO was more beneficial in reducing BPSD. | - Small sample size limits generalizability. - Care staff interviewed were all animal lovers, which may bias results. |
| **5** | Liang A et al., 2017 [7] | New Zealand | Multi | 30 CRs + 30 CGs | 67–98 (CR); 30–86 (CG) | 64 (CR); 96 (CG) | Dementia | PARO | - Investigate the affective, social, behavioral, and physiological effects of PARO in both day care and home settings. | - PARO shows promise in enhancing affective and social outcomes for certain individuals with dementia in community settings. - No significant physiological effects found. | - Small sample size. - Short duration of the study may limit generalizability. |
| **6** | Joranson N et al., 2016 [8] | Norway | Multi | 53 | 62–95 | 67 | Severe dementia | PARO | - Investigate the effect of PARO-assisted group activities on quality of life in older people with dementia. - Examine how dementia severity relates to intervention outcomes. | - Group activities with PARO had a positive impact on quality of life for participants with severe dementia. - PARO seemed especially effective in reducing tension and improving well-being. | - Small sample size limits generalizability. - Difficulty in blinding participants and assessors. |
| **7** | Thodberg K et al., 2016 [9] | Denmark | Multi | 100 | 79–90 | 69 | Dementia | PARO | - Compare behavioral responses of nursing home residents to visits from a person with a dog, robot seal, or toy cat. - Explore whether interaction differs based on animal type and cognitive function. | - Dogs and robot seals prompt more interaction than toy cats. - Residents with higher cognitive impairment engaged more with animals. - Robot seals lost effectiveness over time compared to dogs. | - The novelty effect of the robot seal diminished over time. - The study used only large dogs, and results may differ with smaller breeds. |
| **8** | Moyle W et al., 2014 [10] | Australia | Single | 5 | 70–89 | 80 | Dementia | Giraff (TPR) | - Assess the feasibility of using the Giraff telepresence robot for communication. - Evaluate emotional response and engagement in residents with dementia. - Address social isolation in dementia care through technology. | - Giraff robot was feasible for dementia care. - Positive emotional engagement noted. - Families were able to reduce social isolation through the robot. | - Internet connectivity issues. - Small sample size. - Limited duration of the study. |
| **9** | Moyle W et al., 2015 [11] | Australia | Multi | 380 | 60+ | N/A | Dementia | PARO | - Assess the impact of the PARO robot on agitation and mood. - Evaluate engagement and psychotropic drug use. - Compare with Plush-Toy and Usual Care. | - Not found (protocol paper). | - Intervention only conducted in afternoons. - No comparison with live animals. - Limited to 15 weeks of observation. |
| **10** | Robinson H et al., 2015 [12] | New Zealand | Single | 21 | 71–95 | 67 | Not specified | PARO | - Investigate the effects of interacting with the PARO robot on blood pressure. - Measure heart rate changes. | - PARO has positive effects on cardiovascular measures. - Benefits similar to those from live animals. | - Small sample size. - No control group. - Researcher presence may have impacted results. |
| **11** | Gustafsson C et al., 2015 [13] | Sweden | Single | 4 | 82–90 | 50 | Dementia with BPSD | JustoCat | - Explore reactions of individuals with dementia to a robotic cat. - Measure the impact of a robotic cat on agitation and quality of life. - Gather feedback from relatives and caregivers on usability. | - Robotic cat can reduce BPSD and improve quality of life. - Increased communication and emotional interaction. - JustoCat was seen as a reliable tool for interaction and care. | - Small sample size. - Limited number of measurements. - Possible influence of the Hawthorne effect. |
| **12** | Yamada Y et al., 2014 [14] | Czech Republic | Multi | 4,007 | 83 (maen) | 73 | Cognitive impairment, frailty, depression | N/A | - Examine prevalence of sensory impairment in nursing homes. - Assess associations with clinical problems (cognitive impairment, depression, falls, etc.). - Explore the impact of dual sensory impairments. | - Dual sensory impairments significantly worsen health outcomes. - Sensory impairment correlates with frailty, cognitive decline, and depression. | - Cross-sectional design limits causality conclusions. - Facility participation was voluntary, limiting representativeness. - Reliance on interRAI instrument without additional clinical verification. |
| **13** | Moyle W et al., 2019 [15] | Australia | Multi | 138 | 60+ | N/A | Dementia | PARO | - Evaluate PARO's effect on dementia symptoms. - Analyze individual responses to PARO in a LTC setting. - Inform clinical practice and future research. | - One approach does not fit all for managing BPSD. - Regular reassessment and tailoring interventions is crucial. - Ethical considerations in use of PARO due to attachment issues. | - Small sample size for qualitative reflections. - Limited to one geographic area. - Lack of detailed long-term follow-up. |
| **14** | Moyle W et al., 2019 [16] | Australia | Multi | 20 | 20–65 | 75 | Dementia | PARO | - Examine family perceptions of PARO and Plush Toy in dementia care. - Evaluate impact on BPSD and mood. - Explore engagement with robotic vs non-robotic interventions. | - PARO provided comfort and improved social engagement. - Family members preferred interactive robots over static toys. - High cost of PARO limits its use in care facilities. | - Small sample size. - Limited to family members who had prior experience with pets. - The Plush Toy's lack of movement was a major limitation. |
| **15** | Moyle W et al., 2016 [17] | Australia | Single | 5 | 68–98 | 100 | Dementia | CuDDler | - Explore the feasibility of CuDDler for dementia care. - Assess engagement and emotional responses. - Evaluate the impact on agitation. | - CuDDler could help reduce agitation in some participants. - The design needs improvement. - Participant 'J' benefited from CuDDler due to visual agnosia. | - Small sample size. - Technical issues with CuDDler robot. - Limited to one nursing home setting. |
| **16** | Koceski S et al., 2016 [18] | Macedonia | Single | 30 CRs + 5 CGs | 65–78 (CR) | 43 (CR) | No severe disabilities | Self-designed (TPR) | - Investigate the use and acceptance of a telepresence robot by elderly and professional caregivers. - Measure the perceived usefulness and ease of use of the system. | - Caregivers found the system more useful for medical tasks, while elderly participants preferred social functions like video conferencing. - Perceived ease of use was generally positive across groups. | - Short duration of the study. - Limited sample size. |
| **17** | Mitoku K et al., 2016 [19] | Japan | Single | 1,754 | 65+ | 66 | Cognitive impairment, sensory impairments (vision, hearing) | N/A | - Investigate the association between sensory impairments and cognitive impairment. - Assess the relationship between sensory impairments and mortality. | - 70% of elders had either vision or hearing impairment. - Dual sensory impairment was the greatest risk factor for cognitive impairment and increased mortality. | - Limited to LTC recipients in a rural area of Japan. - No objective test for sensory impairments. |
| **18** | Thodberg K et al., 2016 [20] | Denmark | Multi | 124 | 79–90 | 69 | Cognitive impairment, dementia | PARO | - Study the effects of biweekly dog visits on sleep patterns and psychiatric well-being in elderly people. - Compare the effects of dog visits, robot seal visits, and soft toy cat visits. | - Dog visits increased sleep duration temporarily. - No long-term mental or physical health improvements observed. | - Short visit duration (10 minutes). - Results may not generalize beyond nursing home settings. |
| **19** | Bemelmans R et al., 2015 [21] | The Netherlands | Multi | 91 | N/A | 80 | Dementia | PARO | - Evaluate the outcomes of PARO interventions in psychogeriatric care. - Assess short-term effects on psychological functioning and daily care facilitation. | - PARO increased quality of care and life for elderly with dementia. - Therapeutic interventions more effective than care support interventions. | - Limited effect for care support interventions. - Subjectivity in measurements (IPPA). |
| **20** | Claes V et al., 2015 [22] | Belgium | Multi | 245 | 72.45 (maen) | 68 | N/A | N/A | - Explore the attitudes and perceptions of older adults towards contactless monitoring. - Identify socio-demographic factors influencing technology acceptance. - Analyze the functional and financial concerns raised by older adults. | - Willingness to share information with professional caregivers. - Concerns about privacy with informal caregivers. - Preference for government-funded systems. | - Small sample size limits generalizability. - Pre-implementation phase only. Missing data for some questionnaire items. |
| **21** | Broadbent E et al., 2016 [23] | New Zealand | Multi | 52 | 66–97 | 77 | Not specified | Cafero (TPR), Guide (TPR) | - Assess benefits and problems of healthcare robots in aged care. - Compare quality of life outcomes in intervention and control groups. - Investigate staff and resident perceptions of the robots. | - Robots did not significantly impact quality of life. - Job satisfaction increased only in the control group. - Residents used robots sparingly, and staff perceived robots as having limited benefits. | - Low engagement from residents. - Short duration of the trial. - Limited operational role for the robots. |
| **22** | Rantanen T et al., 2018 [24] | Finland | Multi | 200 | 43 (average) | 94 | N/A | N/A | - Examine attitudes of Finnish home care personnel towards the introduction of care robots. - Identify social psychological factors influencing care robot adoption. - Investigate perceived usefulness of care robots in home care tasks. | - Care robots are welcomed for reminder and safety tasks. - Fear of dehumanization and concerns about care robots replacing human care. - Perceived control and workplace norms play significant roles in robot adoption. | - Low response rate (18.2%). - Survey limited to Finnish home care personnel. - Potential non-response bias. |
| **23** | Tang V et al., 2019 [25] | Hong Kong | Single | N/A | N/A | N/A | N/A | N/A | - Develop an I-GCMS. - Monitor real-time biometric data of the elderly. - Support decision-making in care plan modification. | - IoMT facilitates proactive healthcare delivery. - Real-time monitoring and alerts improve operational efficiency. - Case-based reasoning aids in care plan customization. | - Reluctance of elderly to wear IoT devices. - Risk of system blackout. - Dependency on expert knowledge for care plan adaptation. |
| **24** | Rantanen T et al., 2018 [26] | Finland | Multi | 200 | 43 (average) | 94 | N/A | N/A | - Examine adoption of care robots in home care settings. - Analyze Finnish home care personnel’s attitudes towards robots. - Compare negative and positive attitudes using specific scales. | - Negative attitudes towards robots affect willingness to adopt care robots. - Positive attitudes regarding robots' usefulness in safety and independent living enhance readiness. - Combining societal, psychological, and practical care perspectives is essential. | - The study is limited to a Finnish sample. - Small response rate (18.2%) reduces generalizability. - Cultural context might limit the scale's applicability. |
| **25** | Moyle W et al., 2018 [27] | Australia | Multi | 415 | 60+ | 78 | Dementia | PARO | - Explore the effects of PARO on motor activity and sleep patterns. - Use wearable triaxial accelerometer for measurement. | - PARO could assist in managing motor activity in dementia patients. - Limited evidence for improving sleep patterns. | - Poor tolerance of wearable devices by participants. - Significant amount of missing data due to device issues. |
| **26** | Shiomi M et al., 2015 [28] | Japan | Single | 28 | 74 (average) | 50 | Not specified | Autonomous wheelchair robot | - Investigate intention to use autonomous wheelchair robot. - Compare social behaviors with human caregivers. | - Social behaviors in robots increase intention to use. - Comfort and enjoyment were higher with social behaviors. | - Small sample size. - Limited to a single facility in Japan. |
| **27** | Diraco G et al., 2017 [29] | Italy | N/A | 30 | N/A | N/A | N/A | N/A | - Develop a radar smart sensor for elderly monitoring. - Detect cardiorespiratory signals and body movements. - Evaluate the system's fall detection performance. | - UWB radar successfully detects both falls and vital signs. - Unsupervised learning improves detection performance. - The sensor is effective in AAL applications. | - Variability in accuracy with movement. - Tested only on healthy subjects. |
| **28** | Korchut A et al., 2017 [30] | Poland | Multi | 264 | 55–90 | 66 | MCI, Alzheimer’s Disease | N/A | - Identify user needs for robotic assistance. - Classify findings related to robotic requirements. - Provide a basis for future technology development. | - Robots can meet the needs of patients with cognitive impairments. - Surveyed groups highlight emergency response and task reminders as critical. - Future studies needed for long-term interaction analysis. | - Focus on specific regions (Poland, Spain). - Short study period. Limited sample size for broad generalization. |
| **29** | Berridge C, 2016 [31] | United States | Multi | 41 | 65–103 | N/A | Multiple chronic conditions | N/A | - Examine the relationship between privacy and passive monitoring in independent living. - Identify privacy concerns raised by sensor-based systems. - Provide recommendations for privacy management and technology design. | - Privacy concerns affect adoption of monitoring technology. - Social relationships play a role in managing perceived privacy intrusions. - Technological design must accommodate privacy management needs. | - Limited to one metropolitan area. - Could not include residents who never adopted the system due to privacy concerns. - Lack of systematic understanding of data collection and access among participants. |
| **30** | Berridge C, 2017 [32] | United States | Single | 41 | 65–103 | N/A | Chronic conditions, mobility issues, and general aging-related conditions | N/A | - Understand the concerns and perspectives of users, former users, and non-users of passive monitoring systems in low-income independent living. | - Older adults actively resisted passive monitoring systems. | - Lack of interviews with non-adopters. |
| **31** | Machado A et al., 2017 [33] | Brazil | N/A | N/A | N/A | N/A | N/A | N/A | - Present a situation-aware system for AAL. - Enable the prediction of dangerous situations and automatic responses. - Improve traditional systems by combining reactive, proactive, and extensible approaches. | - Combining reactive and proactive actions enhances safety in AAL environments. - Probabilistic reasoning helped in predicting potential risks. | - Limited to a simulated environment. - Real-world validation is needed. |
| **32** | Erebak S et al., 2019 [34] | Turkey | Single | 102 | 19–40 | 42 | N/A | N/A | - Investigate caregivers' trust in anthropomorphic robots in elder care settings. - Examine how trust relates to their intention to work with robots. - Explore preferences for automation levels in robots among caregivers. | - Anthropomorphism does not necessarily lead to increased trust in robots. - Trust in robots is a significant factor in determining intention to collaborate with them. | - Study used images of robots, not real interaction. - Generalizability may be limited due to sample from a single location. |
| **33** | Jones C et al., 2018 [35] | Australia | Multi | 131 | 84 (mean) | 73 | Dementia | PARO | - Develop the EPWDS. - Establish content validity using the Delphi technique. - Evaluate the psychometric properties of the EPWDS. | - EPWDS is a reliable and valid tool for assessing engagement in people with dementia. - Positive associations between EPWDS and environmental stimulation. | - Results are based on theoretical validation by experts. - Further testing in clinical settings is needed to confirm external validity. |
| **34** | Al-Khafajiy M et al., 2019 [36] | United Kingdom | N/A | N/A | N/A | N/A | N/A | N/A | - Develop a smart healthcare monitoring system for elderly people using wearable sensors. - Enable real-time monitoring to aid early intervention practices. - Improve clinical decision support by providing accurate, low-latency health data. | - Real-time monitoring systems for elderly health can significantly improve early intervention and reduce hospitalizations. - The proposed system is scalable, reliable, and cost-effective. | - Only simulation-based results were obtained. - No real-world testing has been performed. |
| **35** | Rafferty J et al., 2017 [37] | United Kingdom | N/A | N/A | N/A | N/A | N/A | N/A | - Improve assistive smart homes using intention recognition. - Evaluate the system in real-world and simulated environments. - Enhance scalability, reusability, and privacy in smart home applications. | - Intention recognition enhances the functionality of assistive smart homes. - System offers privacy protection by minimizing data collection. | - Sensor placement affected accuracy in real-world scenarios. - Further testing required in diverse environments. |
| **36** | Lloret J et al., 2015 [38] | Spain | N/A | N/A | N/A | N/A | N/A | N/A | - Develop a smart communication architecture for AAL using AI. - Implement a system for monitoring elderly people in real-time. - Enhance scalability, low energy consumption, and ease of use. | - AAL systems are capable of monitoring elderly behavior effectively using AI. - System enhanced decision-making for caregivers and medical staff. | - No real-world testing; results are based on simulations. - Further integration with more sensors and devices required. |
| **37** | Decerle J et al., 2019 [39] | France | N/A | N/A | N/A | N/A | N/A | N/A | - Develop a memetic algorithm to optimize the home health care problem. - Maximize the quality of service, minimize the total working time, and balance the workload among caregivers. - Conduct comparative analysis using benchmark instances. | - A memetic algorithm is effective for multi-objective home health care optimization. - The method helps decision-makers choose optimal trade-off solutions. | - Limited to computational results; no real-world implementation. - Further research required for more diverse instances and real-life testing. |
| **38** | Pfadenhauer M et al., 2015 [40] | Austria, Germany | N/A | N/A | N/A | N/A | N/A | N/A | - Explore how social robots are deployed in dementia care. - Examine the interaction between care workers and the robot PARO. - Investigate the performative role of technology in dementia care. | - PARO facilitated communication and engagement in dementia care. - Care workers developed performative strategies to enhance interaction with PARO. | - Limited to a single care facility in Germany. - No longitudinal data on the long-term impact of PARO on patients. |
| **39** | Cavallo F et al., 2014 [41] | Italy, Belgium | N/A | N/A | N/A | N/A | N/A | N/A | - Explore the interdisciplinary nature of human-machine interaction in socially believable robots. - Analyze the modeling of emotions, behaviors, and contexts in robotic and ICT systems. - Investigate new mathematical models and cognitive architectures for context-aware systems. | - Context-aware systems are critical for creating socially believable robots. - Current systems need further cognitive and computational advancements to meet societal demands. | - Limited real-world testing of theoretical models. - More extensive interdisciplinary collaboration is needed. |
| **40** | Nikoloudakis Y et al., 2016 [42] | Greece, Cyprus, Romania | N/A | N/A | N/A | N/A | N/A | N/A | - Develop a decentralized, virtual fog layer system for emergency alerts in AAL. - Enable location tracking and emergency communication for activity-challenged individuals. - Minimize network latency and enhance system performance using fog computing. | - Fog computing significantly reduces latency and enhances emergency system reliability. - System supports fast response to emergencies in AAL environments. | - No real-world testing; results are simulation-based. - System relies on LoST protocol and geolocation services for performance. |
| **41** | Atee M et al., 2017 [43] | Australia, Kosovo | Multi | 40 | 60–98 | 70 | Dementia | N/A | - Evaluate the psychometric properties of the ePAT compared to the Abbey Pain Scale. - Improve pain detection in people with moderate to severe dementia using facial recognition technology. | - ePAT offers a valid and reliable method to assess pain in dementia patients. - Facial recognition technology automates part of the pain assessment, reducing subjectivity. | - Small sample size and non-random participant selection. - Over-representation of Caucasian females. - Unequal number of assessments per resident. |
| **42** | Pigini L et al., 2017 [44] | Italy | Single | 32 | 53–81 | N/A | Cardiac disorders | N/A | - Develop and test a personal health system integrating environmental and wearable sensors for telemonitoring elderly patients at home. - Improve home healthcare while reducing costs and enhancing quality of life. - Evaluate the reliability and user perception of the system. | - The system can potentially improve home healthcare management for elderly patients. - Clinicians found the system useful but suggested improvements for the clinical interface. | - Small sample size and limited testing of sensor integration. - Further technical refinements needed for larger-scale implementation. |
| **43** | Jones C et al., 2018 [45] | Australia | Multi | 138 | 84 84 (mean) | 73 | Dementia | PARO | - Explore whether cognitive impairment and agitation predict outcomes in engagement, mood, and agitation after 10-week PARO intervention. - Determine the effectiveness of PARO in managing agitation and improving behavioral outcomes. | - PARO is more effective in residents with lower baseline agitation and higher cognitive function. - Severe agitation reduces the effectiveness of the PARO intervention. | - Only a small proportion of variance in outcomes was explained by cognitive impairment and agitation. - The study did not account for individual variability in responses to PARO. |
| **44** | Lehoux P et al., 2018 [46] | Canada | N/A | N/A | N/A | N/A | N/A | N/A | - Explore public perceptions of assistive robots in independent living for older adults. - Investigate the ethical implications of delegating care tasks to robots. | - Robots can supplement but not replace human caregivers. - Delegation of care tasks to robots is limited due to trust and emotional aspects of caregiving. | - Small sample size. - Study focused on participants from a specific region (Quebec). - Mostly educated participants. |
| **45** | Mulero R et al., 2018 [47] | Italy | Multi | 20 | N/A | N/A | MCI, frailty | N/A | - Develop IoT-based solutions for elderly care. - Utilize unobtrusive data gathering and LOD-based management for smart cities. | - IoT and LOD are effective for elderly monitoring. - System scalability and reliability confirmed. - Unobtrusive data gathering worked as intended. | - Testing performed in simulated environments. - Not fully prepared for real-world deployment. |
| **46** | Darragh M et al., 2017 [48] | New Zealand | Multi | 9 | 54–78 | 56 | MCI, Mild Dementia | N/A | - Design a homecare robot for functional support and therapeutic intervention in MCI. - Gather requirements from patients, carers, and experts. | - Robots can help with routines, provide reassurance, track health and well-being. - Robots can improve outcomes for mildly cognitively impaired individuals. | - Small sample size of patients. - No real-world deployment yet. |
| **47** | D'Onofrio G et al., 2019 [49] | Italy | N/A | 17 CRs + 36 CGs | 63–92 (CR); 33–68 (CG) | 53 (CR and CG) | MCI, normal aging | N/A | - Explore the needs of elderly users and caregivers regarding social robots. - Identify priority areas for robot development. - Investigate cross-cultural perceptions of robot usage. | - Robots can address socialization and loneliness issues. - Cross-cultural differences affect robot design preferences. | - Small sample size. - Cultural differences may limit generalization. |
| **48** | Khosla R et al., 2021 [50] | Australia | N/A | 5 | 75–85 | N/A | Dementia | Betty | - Study the engagement of older people with dementia with social robots. - Understand robot experience in a home care setting. - Evaluate the long-term adoption of social robots in dementia care. | - Social robots can enhance engagement and independence in dementia care. - Person-centered design and personalization are critical for long-term engagement. - Emotional engagement and human-like attributes in robots are essential for success. | - Limited to 5 participants, restricting broader generalization. - No audio recording due to privacy concerns. - Negative emotional responses not deeply analyzed. |
| **49** | Cortellessa G et al., 2018 [51] | Italy | N/A | 25 | 37 (mean) | 48 | N/A | ROBIN (TPR) | - Develop a telepresence robot (ROBIN) for older adults. - Assess its capabilities in supporting social inclusion and health monitoring. | - ROBIN effectively supports social inclusion. - Communication services and interaction modalities are key for user satisfaction. | - Small sample size. - No real-world deployment outside lab setting. |
| **50** | Wright J, 2019 [52] | France | Single | 80 CRs + 37 CGs | 89 (mean, CR); 44 (mean, CG) | N/A | Dementia, significant physical disabilities | Pepper, Hug, Paro | - Explore the use of robots in institutional eldercare in Japan. - Investigate whether robots can substitute for human labor in caregiving. - Examine the implications of using robots on human caregivers and the workforce. | - Robots are unlikely to replace human caregivers in the foreseeable future. - Robots reconfigured care and altered the roles of human caregivers, increasing the distance between caregivers and care recipients. - Robots could help overcome linguistic and cultural barriers in caregiving. | - Limited to a single care home. - Observations focus more on caregivers than residents. - Ethical concerns about consent from elderly residents with dementia were noted. |
| **51** | Lukasik S et al., 2020 [53] | Poland | Single | N/A | N/A | N/A | N/A | N/A | - Examine opinions of medical and nursing students on assistive robots. - Understand their perspective on robots' roles in elderly care. | - Both medical and nursing students viewed robots positively for elderly care. - Students believed robots should provide assistance rather than companionship. - Training older people in robot usage is necessary. | - Students had no practical experience with robots. - Small sample size. - Limited to one institution. |
| **52** | Pouyet V et al., 2015 [54] | France | Multi | 104 | 79–101 | 81 | Dementia | N/A | - Investigate the relationship between food liking and food intake in older adults with differing cognitive status. - Study the influence of flavour enhancement on food liking and food intake. - Compare results between paired comparison and sequential monadic tests. | - Flavour enhancement increases food intake regardless of cognitive status. - Sequential monadic testing is as effective as paired comparison testing. - Sensory variety affects food intake, especially in participants with higher cognitive status. | - Sample limited to four nursing homes. - Small sample size. - Cognitive abilities may affect responses to food liking questions. |
| **53** | Wittich W et al., 2018 [55] | Canada | N/A | N/A | N/A | N/A | N/A | N/A | - Identify tools and strategies for sensory screening in older adults with dementia. - Explore barriers and facilitators in implementing these strategies. - Provide recommendations for LTC staff. | - Flexible screening strategies are needed. - Importance of interprofessional collaboration. - Speech-based and personalized stimuli were effective for screening. | - Small sample size (n=11). - Limited to Canadian specialists. - Focus on sensory healthcare specialists, excluding other stakeholders. |
| **54** | Yamada Y et al., 2015 [56] | Czech Republic | Multi | 1,524 | 66–87 | 75 | Dementia, cognitive impairment | N/A | - Investigate whether DSI is associated with the onset of behavioral symptoms in nursing homes. - Examine the relationship between the severity of DSI and behavioral symptoms. - Assess new behavioral symptoms in residents over a 12-month period. | - DSI plays a significant role in the development of behavioral symptoms in nursing home residents. - The severity of sensory impairment did not significantly change the association with behavioral symptoms. - Attention to DSI is important for managing behavioral issues in care settings. | - Small sample of nursing homes. - Possible underreporting of symptoms. - The exclusion of residents with very severe cognitive impairment may limit generalizability. |
| **55** | Daniele K et al., 2019 [57] | Italy | N/A | 50 | 78 (mean) | 76 | Prefrail older adults | Giraff (TPR) | - Understand how prefrail older people perceive ICT. - Explore preferences for robot functionalities in supporting independent living. | - Older adults are open to using robots for support with daily activities. - Preconceptions about technology do not prevent actual use. - The role of robots in assisting with health and safety was particularly valued. | - Small sample size. - Participants were mostly socially active, limiting generalizability. - All participants were from an urban setting in Italy. |
| **56** | Yu R et al., 2014 [58] | Hong Kong | Single | 2,000 | 72 (mean) | 0 | Osteoporosis, Sarcopenia | N/A | - Examine the effects of sarcopenia on incident fractures. - Evaluate the incremental value of sarcopenia for predicting fracture risk. - Assess the combined effects of osteoporosis and sarcopenia on fracture risk. | - Sarcopenia is an independent predictor of fracture risk. - Provides incremental predictive value. - Sarco-osteoporosis subgroup had the highest risk. | - Cohort more educated and physically active than general population. - Findings not generalizable to frail or institutionalized individuals. |
| **57** | Chetty G et al., 2015 [59] | Australia | N/A | 30 | 19–48 | N/A | N/A | N/A | - Develop a scheme for human activity recognition using smartphone inertial sensors. - Evaluate various machine learning techniques. - Enhance activity recognition for eHealth applications. | - Random forests and ensemble methods are effective for activity recognition. - Dimensionality reduction enhances performance. - Suitable for real-time health monitoring applications. | - High-dimensionality affects real-time implementation. - Further testing needed on hardware-constrained devices. |
| **58** | Arends J et al., 2018 [60] | The Netherlands | Multi | 28 | 15–67 | 64 | Epilepsy | N/A | - Develop and evaluate a multimodal seizure detection system. - Compare detection performance to standard bed sensors. - Evaluate usability by caregivers. | - Multimodal sensors provide reliable seizure detection. - Better sensitivity than standard bed sensors. - Suitable for long-term monitoring. | - Excluded patients with movement disorders. - Did not use EEG as gold standard. - Signal quality issues in some cases. |
| **59** | Lazarou I et al., 2016 [61] | Greece | Multi | 4 | 69–80 | 50 | Cognitive impairment, dementia | N/A | - Develop a remote monitoring system for elders with cognitive impairment. - Design personalized interventions. - Improve quality of life and cognitive functions. | - System supports clinicians in adaptive interventions. - Sleep and daily living monitoring provide valuable insights. - Enhances quality of life for participants. | - Small sample size. - Cost of deployment not optimized for scale. - Limited to specific cognitive impairments. |
| **60** | Meritam P et al., 2018 [62] | Denmark | Multi | 71 | 7–72 | 45 | Epilepsy | N/A | - Evaluate the applicability of a wearable seizure detection device. - Assess usability and user satisfaction in the home environment. - Compare sensitivity and false alarm rates with previous studies. | - Device was effective for detecting seizures in home settings. - False alarm rate acceptable to most users. - Device resulted in fewer seizure-related injuries in 40% of cases. | - Small sample size. - Study relied on patient/caregiver self-reporting. - Some users experienced device-related side effects. |
| **61** | Schwenk M et al., 2014 [63] | United States, Germany | Single | 77 | 82 (mean) | 23 | Dementia | N/A | - Explore the validity of sensor-derived physical activity parameters for predicting future falls in people with dementia. - Compare sensor-based fall risk assessment with conventional fall risk measures. | - Sensor-derived physical activity parameters are independent predictors of fall risk in dementia patients. - Telemonitoring technology offers the potential to improve fall prediction. - Results should be confirmed in a larger study. | - Small sample size. - 24-hour monitoring period may not capture day-to-day variability. - Longer-term follow-up needed. |
| **62** | Martinikorena I et al., 2016 [64] | Spain | Single | 24 | 93 (mean) | 75 | Frailty, Sarcopenia | N/A | - Assess the relationship between muscle quality and gait performance. - Evaluate gait variability and its association with muscle power. - Investigate muscle quality and strength in relation to functional outcomes. | - Muscle power and quality are key predictors of gait performance in frail elderly. - Gait variability linked to increased fall risk. - Important for designing interventions to improve stability. | - Small sample size. - Lack of long-term follow-up. - No analysis of cognitive function impact on gait. |
| **63** | Ho A, 2020 [65] | Canada | N/A | N/A | N/A | N/A | N/A | N/A | - Explore the readiness of AI health monitoring for elder care. - Discuss the ethical implications of AI health monitoring. - Examine the balance between safety, privacy, and relational care. | - AI health monitoring has potential but requires ethical considerations. - Stakeholder engagement is crucial for successful implementation. - More empirical research needed on practical applications. | - Lack of empirical data on AI's long-term impact in elder care. - Limited discussion on economic feasibility. - Ethical frameworks need further development. |
| **64** | Lattanzio F et al., 2014 [66] | Italy | N/A | N/A | N/A | N/A | N/A | N/A | - Highlight the role of advanced technology in promoting health and wellbeing among the elderly. - Analyze recent technological developments in Italy and Europe. - Discuss the intersection of geriatric research and technology innovation. | - Italy is a leader in developing healthcare technologies for the elderly. - Telemedicine and robotics are key areas of innovation. - Collaboration between various sectors enhances care outcomes for the elderly. | - Lack of long-term studies on the efficacy of these technologies. - Limited data on cost-effectiveness. - Gaps in empirical research on user experience. |
| Abbreviations: AAL = Ambient-assisted living; BPSD = Behavioral and psychological symptoms of dementia; C/R (CA) = Country/Region (corresponding author); DSI = Dual sensory impairment; ePAT = electronic Pain assessment tool; %F = Female percentage; I-GCMS = IoMT-based geriatric care management system; ICT = Information and communication technology; IPPA = Individually prioritized problems assessment; IoMT = Internet of medical things; IoT = Internet of things; ITP = Illness type of participants; LoST = Location-to-service translation; LTC = Long-term care; LOD = Linked open data; MCI = Mild cognitive impairment; N = Number of total participants; N/A = Not applicable or not available; PWDS = Engagement of a person with dementia scale; SAR = Socially assistive robots; S/M-center = Single/Multi-center; TPR = Telepresence robot; UWB = Ultra-wideband | | | | | | | | | | | |

References

1. Zupic I, Čater T. Bibliometric Methods in Management and Organization. Organizational Research Methods. 2014;18(3):429-72. doi: 10.1177/1094428114562629.

2. Mavragani A, Ochoa G, Tsagarakis KP. Assessing the Methods, Tools, and Statistical Approaches in Google Trends Research: Systematic Review. J Med Internet Res. 2018 Nov 6;20(11):e270. PMID: 30401664. doi: 10.2196/jmir.9366.

3. Joranson N, Pedersen I, Rokstad AM, Ihlebaek C. Effects on Symptoms of Agitation and Depression in Persons With Dementia Participating in Robot-Assisted Activity: A Cluster-Randomized Controlled Trial. J Am Med Dir Assoc. 2015 Oct 1;16(10):867-73. PMID: 26096582. doi: 10.1016/j.jamda.2015.05.002.

4. Moyle W, Jones CJ, Murfield JE, Thalib L, Beattie ERA, Shum DKH, et al. Use of a Robotic Seal as a Therapeutic Tool to Improve Dementia Symptoms: A Cluster-Randomized Controlled Trial. J Am Med Dir Assoc. 2017 Sep 1;18(9):766-73. PMID: 28780395. doi: 10.1016/j.jamda.2017.03.018.

5. Pino M, Boulay M, Jouen F, Rigaud AS. "Are we ready for robots that care for us?" Attitudes and opinions of older adults toward socially assistive robots. Front Aging Neurosci. 2015;7:141. PMID: 26257646. doi: 10.3389/fnagi.2015.00141.

6. Moyle W, Bramble M, Jones C, Murfield J. Care staff perceptions of a social robot called Paro and a look-alike Plush Toy: a descriptive qualitative approach. Aging Ment Health. 2018 Mar;22(3):330-5. PMID: 27967207. doi: 10.1080/13607863.2016.1262820.

7. Liang A, Piroth I, Robinson H, MacDonald B, Fisher M, Nater UM, et al. A Pilot Randomized Trial of a Companion Robot for People With Dementia Living in the Community. J Am Med Dir Assoc. 2017 Oct 1;18(10):871-8. PMID: 28668664. doi: 10.1016/j.jamda.2017.05.019.

8. Joranson N, Pedersen I, Rokstad AM, Ihlebaek C. Change in quality of life in older people with dementia participating in Paro-activity: a cluster-randomized controlled trial. J Adv Nurs. 2016 Dec;72(12):3020-33. PMID: 27434512. doi: 10.1111/jan.13076.

9. Thodberg K, Sørensen LU, Videbech PB, Poulsen PH, Houbak B, Damgaard V, et al. Behavioral Responses of Nursing Home Residents to Visits From a Person with a Dog,a Robot Seal or aToy Cat. Anthrozoös. 2016;29(1):107-21. doi: 10.1080/08927936.2015.1089011.

10. Moyle W, Jones C, Cooke M, O'Dwyer S, Sung B, Drummond S. Connecting the person with dementia and family: a feasibility study of a telepresence robot. BMC Geriatr. 2014 Jan 24;14:7. PMID: 24456417. doi: 10.1186/1471-2318-14-7.

11. Moyle W, Beattie E, Draper B, Shum D, Thalib L, Jones C, et al. Effect of an interactive therapeutic robotic animal on engagement, mood states, agitation and psychotropic drug use in people with dementia: a cluster-randomised controlled trial protocol. BMJ Open. 2015 Aug 12;5(8):e009097. PMID: 26270953. doi: 10.1136/bmjopen-2015-009097.

12. Robinson H, MacDonald B, Broadbent E. Physiological effects of a companion robot on blood pressure of older people in residential care facility: a pilot study. Australas J Ageing. 2015 Mar;34(1):27-32. PMID: 24373064. doi: 10.1111/ajag.12099.

13. Gustafsson C, Svanberg C, Mullersdorf M. Using a Robotic Cat in Dementia Care: A Pilot Study. J Gerontol Nurs. 2015 Oct;41(10):46-56. PMID: 26488255. doi: 10.3928/00989134-20150806-44.

14. Yamada Y, Vlachova M, Richter T, Finne-Soveri H, Gindin J, van der Roest H, et al. Prevalence and correlates of hearing and visual impairments in European nursing homes: results from the SHELTER study. J Am Med Dir Assoc. 2014 Oct;15(10):738-43. PMID: 24984787. doi: 10.1016/j.jamda.2014.05.012.

15. Moyle W, Jones C, Murfield J, Thalib L, Beattie E, Shum D, et al. Using a therapeutic companion robot for dementia symptoms in long-term care: reflections from a cluster-RCT. Aging Ment Health. 2019 Mar;23(3):329-36. PMID: 29282989. doi: 10.1080/13607863.2017.1421617.

16. Moyle W, Bramble M, Jones CJ, Murfield JE. "She Had a Smile on Her Face as Wide as the Great Australian Bite": A Qualitative Examination of Family Perceptions of a Therapeutic Robot and a Plush Toy. Gerontologist. 2019 Jan 9;59(1):177-85. PMID: 29165558. doi: 10.1093/geront/gnx180.

17. Moyle W, Jones C, Sung B, Bramble M, O’Dwyer S, Blumenstein M, et al. What Effect Does an Animal Robot Called CuDDler Have on the Engagement and Emotional Response of Older People with Dementia? A Pilot Feasibility Study. International Journal of Social Robotics. 2015;8(1):145-56. doi: 10.1007/s12369-015-0326-7.

18. Koceski S, Koceska N. Evaluation of an Assistive Telepresence Robot for Elderly Healthcare. J Med Syst. 2016 May;40(5):121. PMID: 27037685. doi: 10.1007/s10916-016-0481-x.

19. Mitoku K, Masaki N, Ogata Y, Okamoto K. Vision and hearing impairments, cognitive impairment and mortality among long-term care recipients: a population-based cohort study. BMC Geriatr. 2016 May 27;16:112. PMID: 27233777. doi: 10.1186/s12877-016-0286-2.

20. Thodberg K, Sorensen LU, Christensen JW, Poulsen PH, Houbak B, Damgaard V, et al. Therapeutic effects of dog visits in nursing homes for the elderly. Psychogeriatrics. 2016 Sep;16(5):289-97. PMID: 26510632. doi: 10.1111/psyg.12159.

21. Bemelmans R, Gelderblom GJ, Jonker P, de Witte L. Effectiveness of Robot Paro in Intramural Psychogeriatric Care: A Multicenter Quasi-Experimental Study. J Am Med Dir Assoc. 2015 Nov 1;16(11):946-50. PMID: 26115817. doi: 10.1016/j.jamda.2015.05.007.

22. Claes V, Devriendt E, Tournoy J, Milisen K. Attitudes and perceptions of adults of 60 years and older towards in-home monitoring of the activities of daily living with contactless sensors: an explorative study. Int J Nurs Stud. 2015 Jan;52(1):134-48. PMID: 24951084. doi: 10.1016/j.ijnurstu.2014.05.010.

23. Broadbent E, Kerse N, Peri K, Robinson H, Jayawardena C, Kuo T, et al. Benefits and problems of health-care robots in aged care settings: A comparison trial. Australas J Ageing. 2016 Mar;35(1):23-9. PMID: 26364706. doi: 10.1111/ajag.12190.

24. Rantanen T, Lehto P, Vuorinen P, Coco K. The adoption of care robots in home care-A survey on the attitudes of Finnish home care personnel. J Clin Nurs. 2018 May;27(9-10):1846-59. PMID: 29575204. doi: 10.1111/jocn.14355.

25. Tang V, Choy KL, Ho GTS, Lam HY, Tsang YP. An IoMT-based geriatric care management system for achieving smart health in nursing homes. Industrial Management & Data Systems. 2019;119(8):1819-40. doi: 10.1108/imds-01-2019-0024.

26. Rantanen T, Lehto P, Vuorinen P, Coco K. Attitudes towards care robots among Finnish home care personnel - a comparison of two approaches. Scand J Caring Sci. 2018 Jun;32(2):772-82. PMID: 28833309. doi: 10.1111/scs.12508.

27. Moyle W, Jones C, Murfield J, Thalib L, Beattie E, Shum D, et al. Effect of a robotic seal on the motor activity and sleep patterns of older people with dementia, as measured by wearable technology: A cluster-randomised controlled trial. Maturitas. 2018 Apr;110:10-7. PMID: 29563027. doi: 10.1016/j.maturitas.2018.01.007.

28. Shiomi M, Iio T, Kamei K, Sharma C, Hagita N. Effectiveness of social behaviors for autonomous wheelchair robot to support elderly people in Japan. PLoS One. 2015;10(5):e0128031. PMID: 25993038. doi: 10.1371/journal.pone.0128031.

29. Diraco G, Leone A, Siciliano P. A Radar-Based Smart Sensor for Unobtrusive Elderly Monitoring in Ambient Assisted Living Applications. Biosensors (Basel). 2017 Nov 24;7(4):55. PMID: 29186786. doi: 10.3390/bios7040055.

30. Korchut A, Szklener S, Abdelnour C, Tantinya N, Hernandez-Farigola J, Ribes JC, et al. Challenges for Service Robots-Requirements of Elderly Adults with Cognitive Impairments. Front Neurol. 2017;8:228. PMID: 28620342. doi: 10.3389/fneur.2017.00228.

31. Berridge C. Breathing Room in Monitored Space: The Impact of Passive Monitoring Technology on Privacy in Independent Living. Gerontologist. 2016 Oct;56(5):807-16. PMID: 26035900. doi: 10.1093/geront/gnv034.

32. Berridge C. Active subjects of passive monitoring: responses to a passive monitoring system in low-income independent living. Ageing Soc. 2017 Mar;37(3):537-60. PMID: 28239211. doi: 10.1017/S0144686X15001269.

33. Machado A, Maran V, Augustin I, Wives LK, de Oliveira JPM. Reactive, proactive, and extensible situation-awareness in ambient assisted living. Expert Systems with Applications. 2017;76:21-35. doi: 10.1016/j.eswa.2017.01.033.

34. Erebak S, Turgut T. Caregivers’ attitudes toward potential robot coworkers in elder care. Cognition, Technology & Work. 2018;21(2):327-36. doi: 10.1007/s10111-018-0512-0.

35. Jones C, Sung B, Moyle W. Engagement of a Person with Dementia Scale: Establishing content validity and psychometric properties. J Adv Nurs. 2018 May 17;74(9):2227-40. PMID: 29772602. doi: 10.1111/jan.13717.

36. Al-khafajiy M, Baker T, Chalmers C, Asim M, Kolivand H, Fahim M, et al. Remote health monitoring of elderly through wearable sensors. Multimedia Tools and Applications. 2019;78(17):24681-706. doi: 10.1007/s11042-018-7134-7.

37. Rafferty J, Nugent CD, Liu J, Chen L. From Activity Recognition to Intention Recognition for Assisted Living Within Smart Homes. IEEE Transactions on Human-Machine Systems. 2017;47(3):368-79. doi: 10.1109/thms.2016.2641388.

38. Lloret J, Canovas A, Sendra S, Parra L. A smart communication architecture for ambient assisted living. IEEE Communications Magazine. 2015;53(1):26-33. doi: 10.1109/mcom.2015.7010512.

39. Decerle J, Grunder O, Hajjam El Hassani A, Barakat O. A memetic algorithm for multi-objective optimization of the home health care problem. Swarm and Evolutionary Computation. 2019;44:712-27. doi: 10.1016/j.swevo.2018.08.014.

40. Pfadenhauer M, Dukat C. Robot Caregiver or Robot-Supported Caregiving? International Journal of Social Robotics. 2015;7(3):393-406. doi: 10.1007/s12369-015-0284-0.

41. Esposito A, Fortunati L, Lugano G. Modeling Emotion, Behavior and Context in Socially Believable Robots and ICT Interfaces. Cognitive Computation. 2014;6(4):623-7. doi: 10.1007/s12559-014-9309-5.

42. Nikoloudakis Y, Panagiotakis S, Markakis E, Pallis E, Mastorakis G, Mavromoustakis CX, et al. A Fog-Based Emergency System for Smart Enhanced Living Environments. IEEE Cloud Computing. 2016;3(6):54-62. doi: 10.1109/mcc.2016.118.

43. Atee M, Hoti K, Parsons R, Hughes JD. Pain Assessment in Dementia: Evaluation of a Point-of-Care Technological Solution. J Alzheimers Dis. 2017;60(1):137-50. PMID: 28800333. doi: 10.3233/JAD-170375.

44. Pigini L, Bovi G, Panzarino C, Gower V, Ferratini M, Andreoni G, et al. Pilot Test of a New Personal Health System Integrating Environmental and Wearable Sensors for Telemonitoring and Care of Elderly People at Home (SMARTA Project). Gerontology. 2017;63(3):281-6. PMID: 28099965. doi: 10.1159/000455168.

45. Jones C, Moyle W, Murfield J, Draper B, Shum D, Beattie E, et al. Does Cognitive Impairment and Agitation in Dementia Influence Intervention Effectiveness? Findings From a Cluster-Randomized-Controlled Trial With the Therapeutic Robot, PARO. J Am Med Dir Assoc. 2018 Jul;19(7):623-6. PMID: 29656838. doi: 10.1016/j.jamda.2018.02.014.

46. Lehoux P, Grimard D. When robots care: Public deliberations on how technology and humans may support independent living for older adults. Soc Sci Med. 2018 Aug;211:330-7. PMID: 30015242. doi: 10.1016/j.socscimed.2018.06.038.

47. Mulero R, Almeida A, Azkune G, Abril-Jimenez P, Arredondo Waldmeyer MT, Paramo Castrillo M, et al. An IoT-Aware Approach for Elderly-Friendly Cities. IEEE Access. 2018;6:7941-57. doi: 10.1109/access.2018.2800161.

48. Darragh M, Ahn HS, MacDonald B, Liang A, Peri K, Kerse N, et al. Homecare Robots to Improve Health and Well-Being in Mild Cognitive Impairment and Early Stage Dementia: Results From a Scoping Study. J Am Med Dir Assoc. 2017 Dec 1;18(12):1099 e1- e4. PMID: 28974463. doi: 10.1016/j.jamda.2017.08.019.

49. D'Onofrio G, Fiorini L, Hoshino H, Matsumori A, Okabe Y, Tsukamoto M, et al. Assistive robots for socialization in elderly people: results pertaining to the needs of the users. Aging Clin Exp Res. 2019 Sep;31(9):1313-29. PMID: 30560429. doi: 10.1007/s40520-018-1073-z.

50. Khosla R, Chu MT, Khaksar SMS, Nguyen K, Nishida T. Engagement and experience of older people with socially assistive robots in home care. Assist Technol. 2021 Mar 4;33(2):57-71. PMID: 31063044. doi: 10.1080/10400435.2019.1588805.

51. Cortellessa G, Fracasso F, Sorrentino A, Orlandini A, Bernardi G, Coraci L, et al. ROBIN, a Telepresence Robot to Support Older Users Monitoring and Social Inclusion: Development and Evaluation. Telemed J E Health. 2018 Feb;24(2):145-54. PMID: 28771398. doi: 10.1089/tmj.2016.0258.

52. Wright J. Robots vs migrants? Reconfiguring the future of Japanese institutional eldercare. Critical Asian Studies. 2019;51(3):331-54. doi: 10.1080/14672715.2019.1612765.

53. Lukasik S, Tobis S, Kropinska S, Suwalska A. Role of Assistive Robots in the Care of Older People: Survey Study Among Medical and Nursing Students. J Med Internet Res. 2020 Aug 12;22(8):e18003. PMID: 32784187. doi: 10.2196/18003.

54. Pouyet V, Cuvelier G, Benattar L, Giboreau A. Influence of flavour enhancement on food liking and consumption in older adults with poor, moderate or high cognitive status. Food Quality and Preference. 2015;44:119-29. doi: 10.1016/j.foodqual.2015.04.014.

55. Wittich W, Hobler F, Jarry J, McGilton KS. Recommendations for successful sensory screening in older adults with dementia in long-term care: a qualitative environmental scan of Canadian specialists. BMJ Open. 2018 Jan 26;8(1):e019451. PMID: 29374673. doi: 10.1136/bmjopen-2017-019451.

56. Yamada Y, Denkinger MD, Onder G, Finne-Soveri H, van der Roest H, Vlachova M, et al. Impact of dual sensory impairment on onset of behavioral symptoms in European nursing homes: results from the Services and Health for Elderly in Long-Term Care study. J Am Med Dir Assoc. 2015 Apr;16(4):329-33. PMID: 25523284. doi: 10.1016/j.jamda.2014.11.006.

57. Daniele K, Marcucci M, Cattaneo C, Borghese NA, Zannini L. How Prefrail Older People Living Alone Perceive Information and Communications Technology and What They Would Ask a Robot for: Qualitative Study. J Med Internet Res. 2019 Aug 6;21(8):e13228. PMID: 31389341. doi: 10.2196/13228.

58. Yu R, Leung J, Woo J. Incremental predictive value of sarcopenia for incident fracture in an elderly Chinese cohort: results from the Osteoporotic Fractures in Men (MrOs) Study. J Am Med Dir Assoc. 2014 Aug;15(8):551-8. PMID: 24703927. doi: 10.1016/j.jamda.2014.02.005.

59. Chetty G, White M, Akther F. Smart Phone Based Data Mining for Human Activity Recognition. Procedia Computer Science. 2015;46:1181-7. doi: 10.1016/j.procs.2015.01.031.

60. Arends J, Thijs RD, Gutter T, Ungureanu C, Cluitmans P, Van Dijk J, et al. Multimodal nocturnal seizure detection in a residential care setting: A long-term prospective trial. Neurology. 2018 Nov 20;91(21):e2010-e9. PMID: 30355702. doi: 10.1212/WNL.0000000000006545.

61. Lazarou I, Karakostas A, Stavropoulos TG, Tsompanidis T, Meditskos G, Kompatsiaris I, et al. A Novel and Intelligent Home Monitoring System for Care Support of Elders with Cognitive Impairment. J Alzheimers Dis. 2016 Oct 18;54(4):1561-91. PMID: 27636843. doi: 10.3233/JAD-160348.

62. Meritam P, Ryvlin P, Beniczky S. User-based evaluation of applicability and usability of a wearable accelerometer device for detecting bilateral tonic-clonic seizures: A field study. Epilepsia. 2018 Jun;59 Suppl 1:48-52. PMID: 29873828. doi: 10.1111/epi.14051.

63. Schwenk M, Hauer K, Zieschang T, Englert S, Mohler J, Najafi B. Sensor-derived physical activity parameters can predict future falls in people with dementia. Gerontology. 2014;60(6):483-92. PMID: 25171300. doi: 10.1159/000363136.

64. Martinikorena I, Martinez-Ramirez A, Gomez M, Lecumberri P, Casas-Herrero A, Cadore EL, et al. Gait Variability Related to Muscle Quality and Muscle Power Output in Frail Nonagenarian Older Adults. J Am Med Dir Assoc. 2016 Feb;17(2):162-7. PMID: 26577625. doi: 10.1016/j.jamda.2015.09.015.

65. Ho A. Are we ready for artificial intelligence health monitoring in elder care? BMC Geriatr. 2020 Sep 21;20(1):358. PMID: 32957946. doi: 10.1186/s12877-020-01764-9.

66. Lattanzio F, Abbatecola AM, Bevilacqua R, Chiatti C, Corsonello A, Rossi L, et al. Advanced technology care innovation for older people in Italy: necessity and opportunity to promote health and wellbeing. J Am Med Dir Assoc. 2014 Jul;15(7):457-66. PMID: 24836715. doi: 10.1016/j.jamda.2014.04.003.
